# Diverse diagnostic and management approaches for acute rheumatic fever in Australia and New Zealand: findings of a prospective clinical study

**DOI:** 10.1136/bmjopen-2025-099827

**Published:** 2025-07-17

**Authors:** Ruwani Peiris, Rachel Webb, Julie Bennett, Jennifer Yan, Joshua Reginald Francis, Bo Remenyi, Florina Chan Mow, Rachel Burgess, Nigel J Wilson, Alicia Stanley, Laura Francis, Rhonda Holloway, Roxanne Westbury, Shirley Lawrence, Yolanda Hernandez-Gomez, David Broadhurst, Nicole J Moreland, Reuben McGregor, Caroline Motteram, Glenn Pearson, Mark Mayo, Anna P Ralph, Jonathan R Carapetis

**Affiliations:** 1 Global and Tropical Health, Menzies School of Health Research, Tiwi, Northern Territory, Australia; 2 Kidz First Children’s Hospital and Community Health Centre for Youth Health, Auckland, New Zealand; 3 Starship Children’s Health, Auckland, Auckland, New Zealand; 4 Department of Paediatrics, Child and Youth Health, The University of Auckland, Auckland, New Zealand; 5 Department of Public Health, University of Otago, Wellington, New Zealand; 6 Department of Molecular Medicine and Pathology, The University of Auckland School of Medical Sciences, Auckland, New Zealand; 7 Royal Darwin Hospital, Casuarina, Northern Territory, Australia; 8 The Kids Research Institute Australia, Nedlands, Western Australia, Australia; 9 School of Science, Edith Cowan University, Perth, Western Australia, Australia; 10 Perth Children’s Hospital, Nedlands, Western Australia, Australia

**Keywords:** Cardiovascular Disease, PAEDIATRICS, Epidemiology, Public health

## Abstract

**Abstract:**

**Objectives:**

To describe diagnostic and management characteristics of acute rheumatic fever (ARF) among participants in the ‘Searching for a Technology-Driven Acute Rheumatic Fever Test’ study, in order to answer clinical questions and determine epidemiological and practice differences in different settings.

**Design:**

Multisite, prospective cohort study.

**Setting:**

One hospital in northern Australia and two hospitals in New Zealand, 2018–2021.

**Participants:**

143 episodes of definite, probable or possible ARF among 141 participants (median age 10 years, range 5–23; 98% Indigenous).

**Primary and secondary outcome measures:**

Participant characteristics, clinical, biochemical and echocardiographic data were explored using descriptive data. Associations with length of stay were determined using multivariable regression analysis.

**Results:**

ARF presentations were heterogeneous with the most common ARF ‘phenotype’ in 19% of cases being carditis with joint manifestations (polyarthritis, monarthritis or polyarthralgia), fever and PR prolongation. The total proportion of children with carditis was 61%. Australian compared with New Zealand participants more commonly had ARF recurrence (22% vs 0%), underlying RHD (48% vs 0%), possible/probable ARF (23% vs 9%) and were underweight (64% vs 16%). Erythrocyte sedimentation rate (ESR) provided an incremental diagnostic yield of 21% compared with C reactive protein. No instances of RHD were diagnosed among participants in New Zealand. Positive throat Group A Streptococcus culture was more common in New Zealand than in Australian participants (69% vs 3%). Children often required prolonged hospitalisation, with median hospital length-of-stay being 7 days (range 2–66). Significant predictors for length of stay in a multivariable regression model were valve disease (adjusted OR (aOR) 1.56, 95% CI 1.23 to 1.98, p<0.001), requirement for corticosteroids (aOR 1.99, 95% CI 1.22 to 3.26, p=0.007) and higher ESR (aOR 1.4, 95% CI 1.17 to 1.67, p<0.001).

**Conclusions:**

This study provides new knowledge on ARF characteristics and management and highlights international variation in diagnostic and management practice. Differing approaches need to be aligned. Meanwhile, locally specific information can help guide patient expectations after ARF diagnosis.

**STRENGTHS AND LIMITATIONS OF THIS STUDY**

- This is a multicentre prospective study across two countries, which allowed us to study and compare current-day presentations of rheumatic fever, identify variations in illness characteristics and in practice, answer specific questions about the relative diagnostic contributions of different blood tests and report on factors relevant to patients and families such as how long they might expect to stay in hospital after a rheumatic fever diagnosis.
- Cultural guidance is provided by an Australian Aboriginal Governance Council, Māori Governance Group and Pacific Governance Group, supporting Indigenous data sovereignty throughout the project.
- To strengthen confidence in the diagnosis of rheumatic fever, allocation of diagnosis was by consensus from an expert clinical panel with access to all relevant participant clinical data.
- Limitations included the enrolment of participants at different time points from symptom onset, and the completeness of recruitment was impacted by factors including non-consent and the COVID-19 pandemic.

## Introduction

 Acute rheumatic fever (ARF) is an autoimmune complication of Group A Streptococcus (GAS) infection mostly affecting children and adolescents.^1^ ^2^ Carditis progressing to rheumatic heart disease (RHD) is considered the most concerning ARF phenotype, but all forms can cause significant morbidity and disruption for children and their families, including prolonged periods of time in hospital. Despite growing knowledge of regional ARF epidemiology,^3^,^7^ there are limited comparative studies on presentation and management such as the proportion of cases with cardiac involvement, hospital length of stay and anti-inflammatory treatment practices, limiting the ability to give families comprehensive information about what to expect after an ARF diagnosis.^8^ There are also knowledge gaps regarding regional differences in approaches to diagnosis and management.

ARF is a heterogeneous condition posing diagnostic challenges. The varied clinical manifestations of ARF such as carditis, joint involvement and chorea require different management approaches and have different long-term outcomes. Clinical practice guidelines are available,^1 9^ but high-quality evidence underpinning some recommendations is lacking, and management can be inconsistent.^10^

There is no diagnostic test for ARF. Diagnosis is guided by the 2015 American Heart Association (AHA) Jones Criteria comprising clinical and laboratory parameters.^11^ However, there are different criteria for high-risk and low-risk settings and international variations. For example, New Zealand applies the high-risk criteria but with modifications for improved specificity: polyarthralgia is a minor (not major) criterion, monarthritis is not included, and higher cut-offs are applied for erythrocyte sedimentation rate (ESR; ≥50 instead of ≥30 mm/hour), C reactive protein (CRP; ≥50 instead of ≥30 mg/L) and streptococcal serology.^12^ To avoid missing subtle cases, ‘probable’ and ‘possible’ ARF diagnoses are defined in the Australian and New Zealand guidelines.^1^ Probable (highly suspected) ARF is defined as an ‘acute presentation which does not fulfil Jones diagnostic criteria for ARF, missing one major or one minor criterion or lacking evidence of preceding streptococcal infection, but ARF is still considered the most likely diagnosis’, and possible (uncertain) ARF as the same, where ‘ARF is considered uncertain but cannot be ruled out’.^1^ AHA, by contrast, includes only ‘possible ARF’ as a clinical presentation where there is good reason to suspect ARF but criteria are not met due to missing results, limited documentation or unclear clinical features.^11^

The ‘Searching for a Technology-Driven Acute Rheumatic Fever Test’ (START) study^13^ aims to address these diagnostic difficulties and inconsistencies by identifying a biomarker diagnostic signature in participants with ARF compared with control diagnoses (‘healthy’, non-ARF Streptococcal infections, other acute conditions and RHD). Here, using the clinical data obtained from the START study participants, we aim to address knowledge gaps in real-world clinical features, describe diagnostic approaches and management and compare these between Australia and New Zealand. We also aim to determine predictors of hospital length of stay and to provide short-term to mid-term prognostic information for young people diagnosed with ARF and their families.

## Methods

This is a multicentre, prospective cohort study, enrolling children and young people with ARF in Australia and New Zealand. This report adheres to the Strengthening the Reporting of Observational Studies in Epidemiology Statement for reporting observational studies.^14^

### Study setting

Participating sites were the Royal Darwin Hospital, a tertiary hospital in Australia’s Northern Territory; Kidz First Children’s Hospital, a secondary hospital in Auckland, New Zealand; and Starship Children’s Hospital, a quaternary hospital with specialist cardiothoracic services, also in Auckland, New Zealand. These hospitals serve regions with the highest rates of ARF in their respective countries and were chosen to provide diversity in ethnicity and ARF severity.^15 16^

### Indigenous data sovereignty and positionality statement

In keeping with Indigenous data sovereignty principles,^17 18^ an Australian Aboriginal Governance Council (including authors MM, GP and RB), Māori Governance Group and Pacific Governance Group (including author FCM) provided input throughout the project on implementation, data reporting and interpretation.

### Patient and public involvement

The research question was designed following our previous work interviewing patients and community members regarding their information needs around ARF.^19^ Indigenous Australian and New Zealand Māori and Pacific researchers, scientists and clinicians (who are authors on this publication) were involved in the design and implementation of the study, as well as providing cultural leadership and a culturally safe environment for participants. Workshops with patients and consumers, led by the Australian Indigenous Governance Group for the project (led by author GP), have commenced and remain underway, to develop resources for disseminating findings to community members.

### Study population

The START study protocol has been published previously.^13^ START is a multicentre, prospective cohort study that enrolled children and young people with suspected ARF in New Zealand and Australia, from November 2018 to November 2021, and healthy controls. This report includes participants with ARF only. Participants could be enrolled more than once if they presented with an ARF recurrence during the study period, but re-enrolment during the same episode of ARF (ie, within 90 days) was not allowed. Inclusion criteria for ARF cases were children and young adults at Royal Darwin Hospital (aged 5–30 years), Kidz First Hospital (aged 2–20 years) or Starship Hospital (aged 2–20 years), with a diagnosis of suspected or confirmed ARF. Research assistants identified and recruited eligible participants. Written, informed consent was provided by participants, or by guardians with participant assent, if younger than 16 years in New Zealand or younger than 18 years in Australia. In New Zealand, self-identified prioritised ethnicity was applied: people identifying as Māori, regardless of any additional ethnicities, were classified as Māori. Similarly, individuals identifying as Pacific Peoples and any other ethnicities (excluding Māori) were classified as Pacific Peoples.^20^ Exclusion criteria included severe anaemia (a reason to avoid additional blood draws), immunosuppression other than corticosteroids or unstable social situation precluding consent for research.

### Data collection for ARF cases

Data were entered onto paper case record forms and then entered into a MEDRIO electronic database. Data comprised: enrolment site; demographic data; ARF diagnosis likelihood (definite, probable or possible),^1^ ARF episode type (initial or recurrent) and ARF phenotype (Jones major and minor criteria); symptom duration; length of stay; investigation results including laboratory, echocardiogram and ECG findings; and treatment (online supplemental table 1). Antibiotics were grouped by class (penicillins, extended spectrum beta-lactams, cephalosporins, etc). Treatment information was obtained from primary care and hospital records. Receipt of benzathine benzylpenicillin was captured if given in the 28 days prior to admission. For other antibiotics, administration in the 7 days prior to admission was included. Medications given for ARF symptom management and intercurrent conditions were also recorded. Additional detail on medication administration (not captured for the START study) was collected from patient medical records, such as actual number of doses given and discharge prescriptions.

### Diagnostic process

Allocation of diagnosis was by consensus from an expert clinical panel, guided by the 2015 Jones Criteria for high-risk settings,^11^ with the modification of definite, probable and possible ARF categories according to published definitions (with possible and probable collectively grouped as ‘uncertain’ ARF).^1^ The panel, comprising an adult infectious diseases physician, paediatric infectious diseases physician, paediatric cardiologist and two specialist nurses, met regularly to review case record forms and primary data including echocardiogram, ECG, laboratory results, hospital discharge summaries and outpatient letters. At least two panel members, including at least one doctor, were present at each meeting. Where the panel identified discrepancies with the clinical treating team’s diagnosis, the clinical treating team was contacted to discuss the cases.

Diagnostic cut-off values for ESR, CRP, antistreptolysin O (ASO), anti-DNase B (ADB) and PR prolongation were as specified in existing guidelines.^1 11^ PR prolongation was not adjusted for heart rate. The diagnostic cut-off to meet the minor criterion of raised inflammatory markers used was ≥30 mm/hour for ESR and ≥30 mg/L for CRP. Previously published age-specific cut-offs for streptococcal serology^21^ and PR prolongation were used.^1 11^ The diagnosis of acute carditis was based on echocardiography reports and the opinion of the clinical panellists, guided by existing published guidelines.^11 22^ Regurgitation of physiologic or trivial grade was classified as normal.

### Data analyses

Data were extracted from MEDRIO and cleaned in Microsoft Excel. Descriptive statistics and regression analysis were conducted using R for Statistical Computing.^23^ Χ^2^ and Fisher’s exact tests were used to compare categorical data. Data were presented as median and IQR or number and proportion. Length of hospital stay in days, used as a proxy measure of illness severity, was log-transformed for regression analysis. Predictors of length of stay were chosen based on clinical relevance and sufficient data points. Selected predictor variables were age, gender, country, study site, ARF certainty (definite vs probable/possible), ARF recurrence, occurrence of preceding symptoms, severity of valve disease (mitral valve regurgitation, aortic valve regurgitation and mitral stenosis), presence of Jones criteria features (carditis, joint involvement, skin involvement [erythema marginatum or subcutaneous nodules], fever, chorea and PR prolongation), maximum biomarker levels (CRP, ESR, ASO and ADB), culture positivity and administration of corticosteroids. Predictors of hospital length of stay were explored in univariable linear regression analysis; variables significantly associated (p value<0.05) were included in a multivariable linear regression model (except where there was high similarity between variables—eg, country retained, and study site excluded; or ESR retained, and CRP excluded). Associations were expressed as ORs with 95% CIs.

## Results

### Demographics of ARF cases

**Figure 1:**
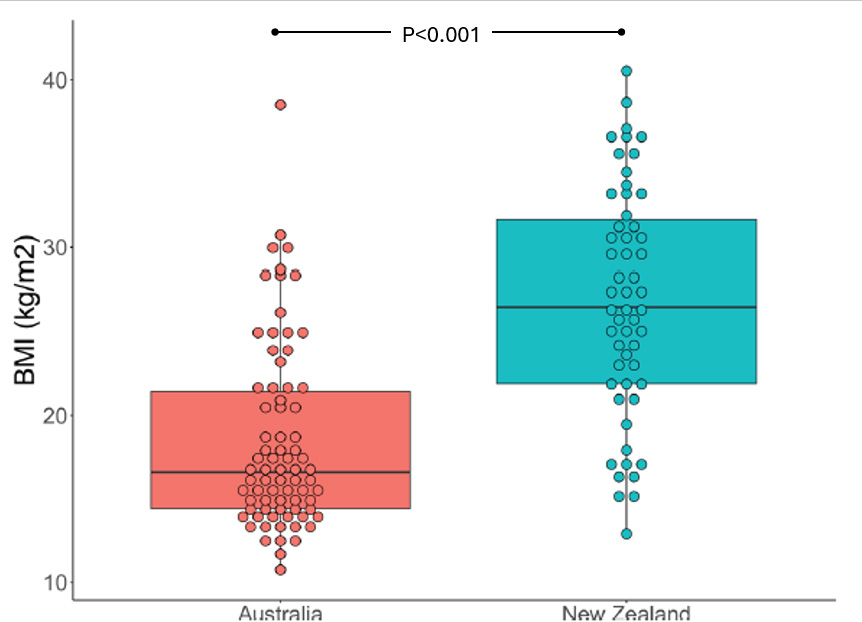
Distribution of body mass index (BMI) of acute rheumatic fever cases, by country.

We recruited 141 participants with 143 presentations where the final diagnosis was ARF; 88 episodes in 86 individuals from Australia and 55 episodes in 55 individuals from New Zealand. Four were outpatients at the time of enrolment following a recent admission (all from Starship Children’s Hospital), the rest were inpatients. Median age was 10 years (IQR 8–12, range 5–23) and 53 (37%) were females. All enrolments in Australia identified as Aboriginal. In New Zealand, 67% identified as Pacific Peoples, 29% as Māori, 4% other (table 1). There was a significant difference in participants’ median body mass index between sites: 16.6 kg/m^2^ (defined as underweight/malnutrition) at Royal Darwin Hospital compared with 28.5 kg/m^2^ at Kidz First and 25.7 kg/m^2^ at Starship Children’s Hospital (both defined as overweight) (p<0.001) (figure 1).

**Table 1:** Characteristics of ARF episodes

Characteristic	Measure	All	Royal Darwin Hospital (AUS)	Kidz First Children’s Hospital (NZ)	Starship Children’s Hospital (NZ)	P value (AUS vs NZ)
Female	n/N (%)	53/143 (37%)	33/88 (38%)	11 (33%)	9/22 (41%)	0.8
Age (years)	Median (range)	10 (5–23)	10/88 (5–23)	10 (5–14)	11 (6–14)	>0.9
BMI (kg/m^2^)	Median (IQR)	20.9 (15.7–27.6)	16.6 (14.4–21.4)	28.5 (23.6–33.3)	25.7 (19.9–29.2)	**<0.001**
Underweight (<18.5 kg/m^2^)	n/N (%)	58/131 (44%)	49/76 (64%)	4/33 (12%)	5/22 (23%)	**<0.001**
Overweight (≥25 kg/m^2^)	n/N (%)	45/131 (34%)	12/76 (16%)	21/33 (64%)	12/22 (55%)	**<0.001**
Ethnicity						
Aboriginal	n/N (%)	88/143 (62%)	88/88 (100%)	–	–	**<0.001**
Māori	n/N (%)	16/143 (11%)	–	10/33 (30%)	6/22 (27%)	
Pacific Peoples	n/N (%)	37/143 (26%)	–	23/33 (70%)	14/22 (64%)	
Other	n/N (%)	3/143 (2%)	–	1/33 (3%)	2/22 (9%)	
Definite ARF	n/N (%)	118/143 (83%)	68/88 (77%)	31/33 (94%)	19/22 (86%)	0.194
Probable ARF	n/N (%)	16/143 (11%)	12/88 (14%)	1/33 (3%)	3/22 (14%)	
Possible ARF	n/N (%)	9/143 (6%)	8/88 (9%)	1/33 (3%)	0	
Initial ARF	n/N (%)	124/143 (87%)	69/88 (78%)	33/33 (100%)	22/22 (100%)	**<0.001**
Recurrent ARF	n/N (%)	19/143 (13%)	19/88 (22%)	0	0	
ARF with RHD	n/N (%)	44/140 (31%)	44/85 (52%)	0	0	**<0.001**
RHD diagnosed this admission	n/N (%)	30/44 (68%)	30/44 (68%)	0	0	
RHD diagnosed previously	n/N (%)	14/44 (32%)	14/44 (32%)	0	0	
LOS (days)		7 (2–66)	7 (2–49)	9 (3–38)	15 (2–66)	**<0.001**
LOS definite ARF (days)	Median (range)	8 (2–66)	7 (2–49)	9 (3–38)	15 (2–66)	
LOS probable ARF (days)	Median (range)	7 (2–14)	7 (2–14)	7 (–)	14 (13–14)*	
LOS possible ARF (days)	Median (range)	3 (2–13)	4 (2–13)	3 (–)	–	

P values were calculated using Pearson’s χ2 test, Kruskal-Wallis rank sum test or Fisher’s exact test.

Bolded values are statistically significant at p<0.05.

* Of the three probable cases at Starship Hospital, length of stay was available for only two.

ARF, acute rheumatic fever; AUS, Australia; BMI, body mass index; LOS, length of stay; N, denominator; NZ, New Zealand; RHD, rheumatic heart disease.

### ARF certainty and ARF recurrence

In Australia, 68 (77%) had definite and 20 (23%) had uncertain (probable or possible) ARF, versus 50 (91%) and 5 (9%), respectively, at the New Zealand hospitals (p=0.043; table 1). ARF recurrence was common in Australia but not reported at all in New Zealand (p<0.001; table 1).

### Rheumatic heart disease

None of the New Zealand participants were considered to have RHD. By contrast, half the Australian participants were labelled as having RHD (one adult in their early twenties, the rest under 18 years) (p<0.001; table 1). These comprised 30 cases of RHD diagnosed concurrently with the index ARF presentation, and 14 cases of RHD diagnosed in the past.

### Clinical manifestations of ARF

**Figure 2:**
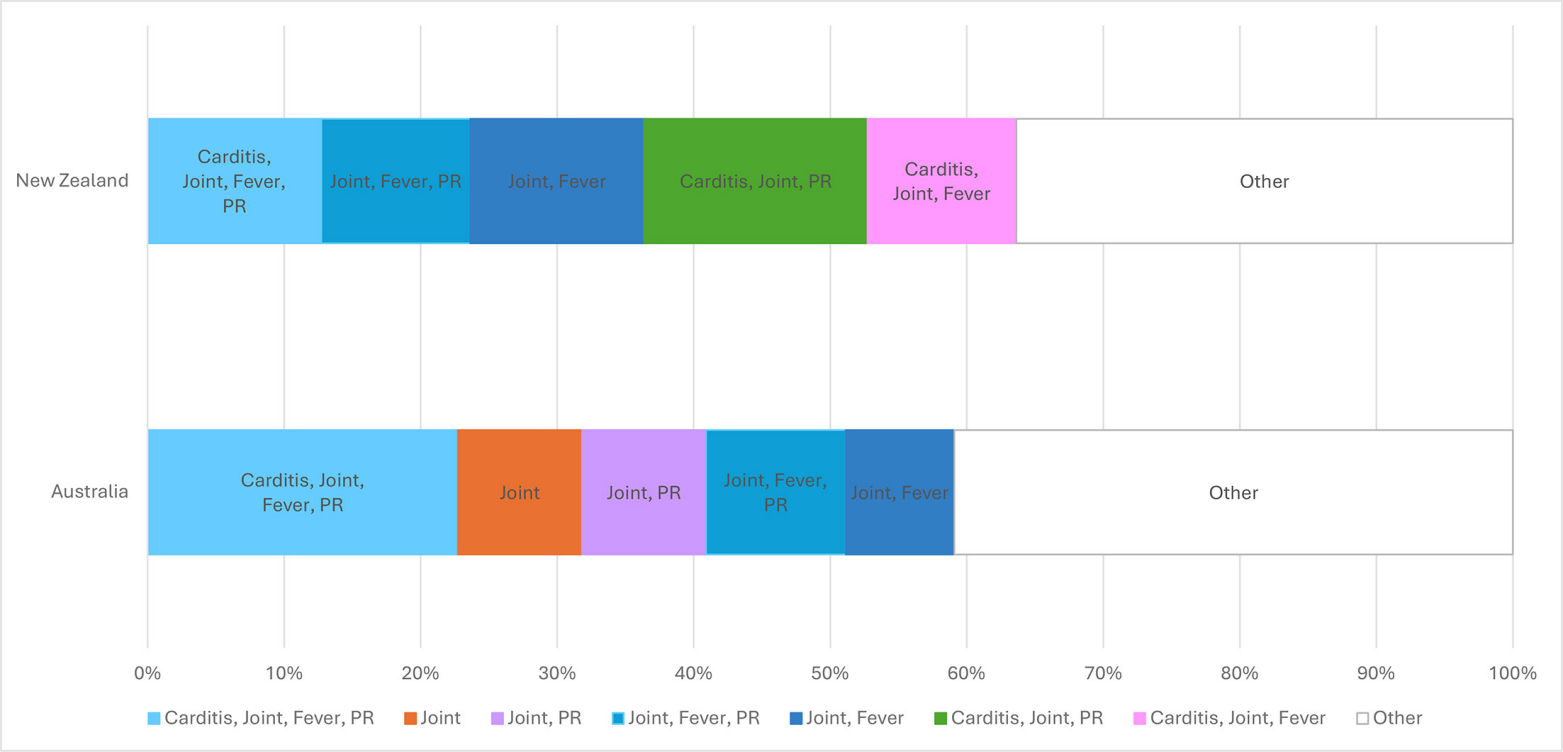
Variation between Australia and New Zealand in top five clinical phenotypes of ARF cases. Carditis, rheumatic valvular abnormality on echocardiogram; Fever, subjective or objective (≥38.0°C) fever; Joint, polyarthralgia, polyarthritis or aseptic monarthritis; PR, PR prolongation; Skin, erythema marginatum.

The most common major criterion was joint involvement (arthritis or arthralgia, 127, 89%), followed by carditis (87, 61%) and chorea (11, 8%) (table 2; online supplemental figure 1). The most frequent clinical phenotype was carditis, joint involvement, fever and prolonged PR interval in 27 episodes (19%) (figure 2). Of 11 children with Sydenham’s chorea, 9 were female and ten had cardiac involvement (eight considered to have RHD and two considered to have acute carditis without chronic changes). Erythema marginatum was reported in seven children, six of whom were New Zealand Pacific peoples, and occurred in combination with joint involvement, with or without other diagnostic criteria. The seventh case was an Aboriginal child with possible ARF due to having erythema marginatum as the only Jones criterion. No participants had subcutaneous nodules detected. First-degree heart block (PR prolongation) was identified in 57%. There was no statistically significant difference in first-degree heart block occurrence in the presence (61%) or absence (52%) of carditis (table 2; online supplemental figure 2).

**Table 2:** Clinical features of ARF cases

	Number (%) or median (IQR)				P value (AUS vs NZ)
	All(n=143)	Royal Darwin Hospital (AUS) (n=88)	Kidz First Children’s Hospital (NZ) (n=33)	Starship Hospital (NZ)(n=22)	
Joint involvement	127 (89%)	77 (88%)	30 (91%)	20 (91%)	0.529
Carditis*	87 (61%)	53 (60%)	21 (64%)	13 (59%)	0.850
Prolonged PR interval	82 (57%)	48 (55%)	19 (58%)	15 (68%)	0.392
In presence of carditis	53/87 (61%)	–	–	–	0.366
In absence of carditis	29/56 (52%)	–	–	–	
Any fever (subjective or documented)	78 (55%)	46 (52%)	16 (48%)	16 (73%)	0.490
Documented fever (≥38.0°C)	53 (37%)	37 (42%)	12 (36%)	4 (18%)	N/A
Chorea	11 (8%)	8 (9%)	3 (9%)	0	0.531
Erythema marginatum	8 (6%)	1 (1%)	3 (9%)	4 (18%)	**0.005**
Subcutaneous nodules	0	0	0	0	–
**Clinical phenotype**					
Carditis, joint, fever, prolonged PR interval	26 (18%)	20 (23%)	5 (15%)	2 (9%)	0.137
Joint, fever, prolonged PR interval	15 (10%)	9 (10%)	3 (9%)	3 (14%)	0.645
Joint, fever	14 (10%)	7 (8%)	4 (12%)	3 (14%)	0.490
Joint, prolonged PR interval	12 (8%)	8 (9%)	3 (9%)	1 (5%)	0.423
Carditis, joint, prolonged PR interval	16 (11%)	7 (8%)	6 (18%)	3 (14%)	**0.037**

Seven ARF cases with carditis additionally had trivial, small or large pericardial effusion. P values were calculated using Pearson’s χ2 test, Fisher’s exact test or Wilcoxon rank sum test.

* The term carditis is used throughout to describe rheumatic valvulitis.

ARF, acute rheumatic fever; AUS, Australia; N, denominator; NZ, New Zealand; RHD, rheumatic heart disease.

### Inflammatory markers

Median values for peak documented CRP and ESR were 65.0 mg/L (IQR 25.3–107) and 82 mm/hour (IQR 35.2–108), respectively (online supplemental table 2). Children with pure chorea without other criteria (n=3) had no elevation in inflammatory markers, but all had significantly elevated ASO and ADB, despite positive serology not being required for Sydenham’s chorea diagnosis. ESR was significantly higher in New Zealand participants (p<0.001; online supplemental table 2).

There were 139 episodes with results for both CRP and ESR (online supplemental table 3). More episodes met the inflammatory marker diagnostic criterion based on elevated ESR than elevated CRP. Among 115 definite ARF episodes, both ESR and CRP were elevated in 88, 19 had elevated ESR only and 1 had elevated CRP only, giving an incremental yield of ESR of 21% (19 out of 89 cases).

### Preceding symptoms of streptococcal infection and primary prophylaxis

Self-reported symptoms of sore throat or skin sores were documented in 79/132 (60%) episodes in the 8 weeks before hospitalisation (online supplemental table 4). 42/75 (56%) participants with preceding sore throat or skin sores recalled receiving antibiotic treatment prior to hospitalisation. Participants in New Zealand were more likely to report preceding symptoms and receipt of antibiotics. For example, 86% of participants at Starship Hospital reported preceding symptoms compared with 54% at Royal Darwin and 58% at Kidz First hospitals (p=0.029; online supplemental table 4).

### Laboratory evidence of preceding streptococcal infection

Throat swabs were collected for culture (on admission or in the preceding 35 days) for nearly all ARF episodes in New Zealand participants (52/55, 97%) compared with just over a third (32/88, 36%) of Australian ARF episodes (online supplemental table 2). Throat culture positivity varied between Kidz First and Starship hospitals (84% vs 45%; p=0.0027) and between the combined New Zealand sites and Royal Darwin Hospital (69% vs 3%; p<0.001).

Streptococcal serology (ASO, ADB or both) was available in 130 cases and found to be elevated in 103/107 (96%) definite ARF, 13/15 (87%) probable ARF and 7/8 (88%) possible ARF episodes (online supplemental tables 2 and 3). ASO and ADB results were both available in 107 definite ARF cases; among these, both markers were elevated in 75 cases; ASO only in 23 and ADB only in 5, highlighting the small incremental added value of testing both (percentage incremental yield of ABD=5%, that is, 5/98).

### Echocardiography findings

87 ARF episodes (61%) had an abnormal echocardiogram reported. These included 82 definite ARF, 5 probable ARF and 1 with a finding of congenital heart disease (online supplemental table 6). Mitral valve regurgitation was the most common valve abnormality (78, 55%), followed by aortic regurgitation (41, 29%), while 32 ARF episodes (22%) had abnormalities of both valves. Nine (6%) had features of mitral stenosis. Seven episodes involved pericardial effusion (six trivial or small, one large); all seven also had rheumatic valvulitis.

There were 44 episodes labelled as having RHD on presentation, among 42 participants (1 adult aged 22, the rest children aged<18 years) (table 1). We identified differences between sites in diagnosing RHD during an acute episode, with the New Zealand approach being to defer such diagnosis until resolution of the acute inflammatory illness. Consequently, all 44 diagnoses of RHD were in Australia, with no New Zealand participants diagnosed with RHD despite mitral stenosis being seen on the echocardiograms of three participants.

Of the 44 with RHD, 30/44 (68%) were diagnosed during the current admission (28 of which comprised a first-recognised episode of ARF) and 14 (32%) were in individuals with known pre-existing RHD. Three participants at Royal Darwin Hospital also had resolved RHD (a prior diagnosis of RHD, but a normal echocardiogram when enrolled into this study).

Echocardiographic abnormalities in longer-standing RHD (diagnosed ≥2 years ago) were generally more severe. For example, mitral regurgitation was moderate or severe in 7/10 (70%) of cases with RHD diagnosed ≥2 years ago, compared with 11/34 (33%) of recent or new RHD diagnoses. Aortic regurgitation was moderate or severe in 3/10 (33%) episodes with long-standing RHD compared with 1/34 (3%) with recently diagnosed RHD.

The single episode of congenital heart disease was characterised by severe mitral regurgitation. This was in a child considered to have definite ARF based on polyarthritis, fever, prolonged PR interval, elevated ESR and CRP and positive streptococcal serology.

### Treatments received

Most episodes had documented receipt of antibiotics (Australia 76/86 (88) vs New Zealand 49/53 (92%); p=0.567) (table 3). These were mostly beta-lactams (online supplemental table 7). A greater proportion of episodes with initial ARF were documented as receiving antibiotics (112/120, 93%) compared with episodes with recurrent ARF (12/18, 67%; p=0.003), noting that all ARF recurrences occurred at the Australian site.

**Table 3:** Medications prescribed for rheumatic fever

Treatment	All		Royal Darwin Hospital (AUS)		Kidz First Hospital (NZ)		Starship Hospital (NZ)	
	N	n (%)	N	n (%)	N	n (%)	N	n (%)
Antibiotics*	141	127 (90%)	86	76 (88%)	33	31 (94%)	22	20 (91%)
Corticosteroids	143	7 (5%)	88	6 (8%)	33	0	22	1 (5%)
NSAIDs	143	65 (45%)	88	43 (49%)	33	14 (42%)	22	8 (36%)
Ibuprofen	65	55 (85%)	43	33 (77%)	14	14 (100%)	8	8 (100%)
Aspirin	65	10 (15%)	43	10 (23%)	14	0	8	0
Naproxen	65	4 (6%)	43	2 (5%)	14	2 (14%)	8	0

* Includes oral or intravenous antibiotics 0–7 days prior to admission or during admission, and intramuscular benzathine benzylpenicillin 0–28 days prior to admission or during admission.

AUS, Australia; N, denominator; NSAIDs, non-steroidal anti-inflammatory drugs; NZ, New Zealand.

Corticosteroid treatment was prescribed in seven ARF episodes: six for carditis, one for chorea; six in Australia, one in New Zealand (online supplemental table 8). Regimens included methylprednisolone for 3 days for one child with rheumatic carditis and complete heart block; and oral prednisolone, for courses of 19–72 days (including one child with symptom rebound on cessation of prednisolone at 13 days, requiring reinstitution for a further 6 days).

65 episodes (45%) were documented as receiving non-steroidal anti-inflammatory drugs (NSAIDs) (table 3). The most commonly prescribed NSAID was ibuprofen (55 cases) followed by aspirin (10 cases, all at the Australian site) and naproxen (4 cases).

Details of NSAID administration were obtained through chart review of 31 Australian and 21 New Zealand episodes. At the Australian site, indications for NSAIDs were joint involvement (29), high fever (1) and unknown (1). Nearly all dosages of ibuprofen were 10 mg/kg or 400 mg per dose, charted regularly in 20 cases and ‘as needed’ in 7 cases. The NSAID duration on discharge was documented in only five episodes, for durations of 5–16 days. The total median inpatient and outpatient duration of ibuprofen prescription among this Australian subset was 4 days (range 1–29 days). At the New Zealand sites, NSAIDs were administered 1–25 days before admission, although two episodes recorded NSAIDs given in the hospital. 19 episodes recorded receipt of ibuprofen, 1 of naproxen and 1 of both.

Medications such as iron supplements and antihelminthics were commonly prescribed for comorbidities among children with ARF, highlighting the illness burden in this population.

### Hospital length of stay and associated factors

Hospitalisation duration varied by site, being longest among 18 patients at Starship Children’s Hospital (median 15 days, range 2–66) and shortest among the 88 patients at Royal Darwin Hospital (median 7 days, range 2–49) (table 1). Definite ARF cases were hospitalised for a median of 8 days (range 2–66) compared with 7 days for probable ARF (range 2–14) and 3 days for possible ARF (range 2–13) (table 1). Carditis occurring simultaneously with other major criteria was associated with the longest hospitalisations (online supplemental table 9). The longest hospitalisation was 66 days in a child with severe carditis, PR prolongation, joint involvement, erythema marginatum and fever. Prolonged hospitalisation of 46 days also occurred in an individual with severe carditis and chorea. Shorter hospitalisation of a week or less was common among participants with joint manifestations without carditis (n=57, length of stay ≤7 days for 67% of these cases).

In univariable analyses, factors significantly associated with longer hospitalisation were presence of valvular pathology (OR 1.89, 95% CI 1.49 to 2.40, p<0.001), enrolment in New Zealand (OR 1.59, 95% CI 1.24 to 2.05, p<0.001), enrolment at Starship Children’s Hospital (OR 2.12, 95% CI 1.49 to 3.02, p<0.001), having definite ARF (OR 1.48, 95% CI 1.18 to 1.87, p=0.001), longer time between symptom onset and enrolment (OR 1.08, 95% CI 1.03 to 1.13, p=0.001), higher peak ESR (OR 1.64, 95% CI 1.38 to 1.94, p<0.001) and higher peak CRP (OR 1.18, 95% CI 1.05 to 1.34, p=0.008) (online supplemental table 10). Receipt of corticosteroids, reserved for severe cases and mostly observed in Australian participants, was also significantly associated with longer hospitalisation (OR 2.48, 95% CI 1.41 to 4.38, p=0.002). In multivariable analysis, higher peak ESR (adjusted OR (aOR) 1.4, 95% CI 1.17 to 1.67, p<0.001), presence of mitral/aortic valve disease (aOR 1.56, 95% CI 1.23 to 1.98, p<0.001) and receipt of corticosteroids (aOR 1.99, 95% CI 1.22 to 3.26, p=0.007) remained associated with length of stay (online supplemental table 11).

## Discussion

In this multisite study, we identified that ARF presentations were similar across the sites, but there were variations in measures of illness severity, streptococcal infection epidemiology, diagnostic practices especially in relation to classification of RHD in the acute setting and variations in management approaches (such as treatment choices and length of hospitalisation). These findings provide opportunities for mutual learning and harmonisation of approaches. Useful information for people diagnosed with ARF and their families includes details on anticipated hospital length of stay, how long NSAIDs and corticosteroids may need to be continued and concomitant comorbidities such as nutritional issues (both overnutrition and undernutrition) that may need to be addressed among children presenting with ARF.

The extreme diversity in ARF symptoms (online supplemental figure 1 and online supplemental table 9 showing 22 different phenotypes) highlights why this condition can be enigmatic for community members and healthcare providers. Permutations of valve and joint involvement, with or without fever and PR prolongation, comprised the most common presentations at both sites. Erythema marginatum was diagnosed more often in New Zealand Pacific Peoples, potentially due to ethnic variation in disease manifestations, ease of detection of this feature in lighter-coloured skin, or variation in local clinician awareness of this uncommon characteristic of ARF.

Hospital length of stay was greatest if significant carditis was present. Moderate or severe carditis occurring with other major criteria was often associated with particularly long hospitalisation requirements of a month or more, while people with joint manifestations as the only major criterion were generally discharged within a week. However, local practice differences also influenced the length of stay. Longer duration of bed rest occurred at Starship Children’s Hospital, which is a tertiary cardiosurgical centre. Prolonged bed rest for rheumatic carditis had been a recognised practice among some practitioners at Starship Children’s Hospital until recently, and the legacy of that practice remains evident. Revised editions of national ARF management guidelines have been published in both New Zealand and Australia in 2025, which respectively recommend early discharge for clinically stable patients and potential hospital avoidance in some selected cases. At Royal Darwin Hospital, about 12% of admissions of Aboriginal people end in self-discharge, which truncates the planned length of stay,^24^,^26^ although this is likely to be more common in adult than paediatric admissions; we do not have data on whether START study participant admissions ended in self-discharge. For these various reasons, hospital length of stay in this study can only partially be considered a measure of disease severity, but the data still provide helpful information to guide discussions with families about likely planned admission times.

Other practice differences were noted at the different study sites: in Australia, penicillin is usually given as intramuscular benzathine benzylpenicillin (BPG) per local guidelines, and usually in the primary care setting as soon as ARF is suspected. In New Zealand, standard practice is to commence oral penicillin and give intramuscular BPG just before discharge from the hospital after the child and family have been well prepared through engagement with a play specialist, multidisciplinary team, and cultural support workers as appropriate. This patient-centred approach should be considered for wider adoption.

Higher rates of recurrent ARF at the Australian site may reflect differences in access to and support for ongoing antibiotic secondary prophylaxis, as well as the risk of exposure to circulating GAS infections. Previous reports indicate that 30–40% of clients in the Northern Territory of Australia receive ≥80% of scheduled BPG injections,^27^ compared with 80–100% in New Zealand.^28 29^

Higher RHD numbers at the Australian site and the absence of RHD from the New Zealand ARF participants may reflect, in part, the higher ARF recurrence rates in Australia, with more cases presenting on a background of previously diagnosed RHD. However, differences in the approach to diagnosis of RHD were also discovered, with the New Zealand approach being to defer such diagnosis until resolution of the acute inflammatory illness unless there are unequivocal chronic changes. Therefore, while none of the New Zealand cases were classified as having RHD in the current study, the three participants with mitral stenosis may have been diagnosed subsequently. Internationally, the diagnosis of RHD has been guided by the 2012 World Heart Federation guidelines^22^ (updated since this study was conducted^30^). As noted in the 2015 AHA Jones criteria update,^11^ the colour-Doppler criteria for pathological valvular regurgitation are the same for carditis of ARF as for mild chronic RHD. However, the morphological valvular features seen in acute carditis overlap but are not identical to those seen with chronic RHD. It is acknowledged that a proportion of patients have improvement in valvulitis, including resolution of morphological changes, in the months after an ARF episode.^31 32^ Chronic RHD is best determined by follow-up echocardiography after resolution of the acute episode. In contrast, the practice at the Australian site has been to apply a diagnosis of RHD acutely, if the echocardiogram findings are considered to constitute RHD.^22^ This practice might relate to the recognised challenge in access to follow-up echocardiography, given the geographical and health service access barriers faced in remote parts of Australia’s Northern Territory. Missing the opportunity to make the diagnosis in the acute setting may mean a true case of RHD is not registered and managed appropriately. However, a potential adverse outcome of this practice is inadvertent diagnosis of RHD in those with carditis that resolves. We are exploring these differences in approaches to RHD diagnosis in ongoing research. Aligning approaches is of major importance to allow valid comparisons of regional and longitudinal epidemiology. However, echocardiography remains a relatively blunt tool in being able to distinguish acute carditis alone from acute carditis occurring on a background of RHD.^30^

These diagnostic uncertainties highlight the need for blood biomarkers not just to improve the accuracy of ARF diagnosis but also to stratify risk of progression.^13^ Subsequent analyses from the START study seeking to identify a diagnostic signature for ARF, and aiming to explore biomarker profiles in different forms of ARF, will address this.

Further notable intersite differences relate to throat swab collection practices and throat swab positivity at the time of ARF diagnosis. Throat swabs were uncommonly done at the Australian setting as part of the routine ARF workup. Of swabs collected, only 3% of throat swabs collected in Australia were positive for GAS, versus 69% of throat swabs in New Zealand. This could be attributed to potential earlier timing of antibiotic administration in Australian participants in relation to when the swab was collected or may reflect known regional epidemiological differences. When surveillance studies have been undertaken in Australia, rates of GAS skin infection generally are more common than pharyngitis,^33^ including during an ARF outbreak,^34^ whereas high rates of both throat and skin infection occur in New Zealand.^35^ Future studies incorporating molecular tests, which have superior sensitivity to culture, may shed more light on streptococcal epidemiology.^36 37^

Australian guidelines on NSAIDs for joint symptom relief recommend their use, indicating that ‘some patients who have persisting joint symptoms may require regular anti-inflammatory therapy for up to 6 weeks’. By contrast, the prescribed duration in the subset of patients we could review in detail was short at one to 29 days, with most less than 2 weeks. A change in guidelines preferring non-aspirin NSAID alternatives was adopted in recent years; there was evidence of uptake of this recommendation, with few children (n=10, all in Australia) being prescribed aspirin. Half of the participants with joint pain (n=127) did not appear to receive NSAIDs (documented as prescribed in 65 cases). While some patients with mild or quickly resolving joint pain may not need NSAIDs, it is important to ensure that all patients are receiving adequate pain relief and that ARF management follows best practice.

Regarding CRP and ESR as a minor criterion for ARF diagnosis, ESR was higher and more likely to be over the diagnostic threshold than CRP, especially for probable and possible cases (online supplemental table 3). However, it remains appropriate to test both, because CRP is more accessible and still adds some value, noting it was elevated in one definite ARF in which ESR was not. ESR testing is inaccessible in remote settings unless blood sample collection can be timed to coincide with air transport to a laboratory within 4 hours of collection and is less available in some New Zealand laboratories than CRP; sometimes ESR can only be done once the patient is in the hospital. Like serological testing, CRP and ESR levels fluctuate throughout the illness with different rates of decay, and our study may not have captured peak levels. When ESR is not available, and a minor criterion is lacking to be able to reach an ARF diagnosis, clinicians should err on the side of caution in considering possible ARF.

We found a small added value of ADB in supporting ARF diagnosis. While ASO was more likely to be elevated than ADB (online supplemental table 5), ADB was elevated in 6 of 13 cases in which ASO was not elevated. ADB is often unavailable as a routine laboratory test, especially in lower-resource settings internationally, but our findings support making this test available for cases with negative ASO and negative streptococcal cultures, where proof of prior infection is needed to make a diagnosis of ARF.

Strengths of this study include its prospective design, the large number of cases given the relative rarity of this condition and the application of the same study protocol at international sites, permitting wider generalisability. Limitations include the variable enrolment time in relation to ARF onset date, leading to potential variation in measures that evolve quickly such as symptoms, inflammatory marker concentration, streptococcal serological titres and ECG changes. We do not present longitudinal outcome data since this was a study of ARF diagnosis, not follow-up. Only a proportion of eligible cases were enrolled, because the recruitment phase coincided with the onset of the COVID-19 pandemic, and because of non-consent or difficulty with the timing of blood collection for the biomarker component of the START study (to be reported elsewhere). We do not believe that there were likely to be systematic differences between enrolled and non-enrolled eligible participants, since the included cases reflect expected demographic and disease severity characteristics and likelihood of ARF recurrence, at the respective study sites.

## Conclusion

This study contributes new information on the clinical presentation of ARF for Australian and New Zealand patients. It provides insights into the length of stay and real-world treatment usage that can be used to inform discussions with patients. We identified unexpected differences in diagnostic and clinical practices, highlighting the need for international harmonisation and development of evidence-based treatment guidelines. Next steps include biomarker analyses to determine whether ARF diagnosis or risk stratification can be improved and the development of multicentre trials to provide stronger evidence to guide ARF management.

## Supplementary material

10.1136/bmjopen-2025-099827 online supplemental file 1

## Data Availability

Data are available upon reasonable request.
