# Supplementary material for: Diverse diagnostic and management approaches for acute rheumatic fever in Australia and New Zealand: findings of a prospective clinical study
Source: BMJ Open. 2025 Jul 17;15(7):e099827. doi: 10.1136/bmjopen-2025-099827 (PMC12273072; doi:10.1136/bmjopen-2025-099827)
Supplement: online supplemental file 1 [file bmjopen-15-7-s001.docx]

Supplementary Material

**Diverse diagnostic and management approaches for acute rheumatic fever in Australia and New Zealand: findings of a prospective clinical study**

Contents

[Table S1. START study data collected for analysis. 2](#_Toc185613388)

[Table S3. CRP and ESR by ARF type. 5](#_Toc185613389)

[Table S4. Preceding symptoms of a relevant infection and receipt of antibiotics 6](#_Toc185613390)

[Table S5. ASO and anti-DNaseB by ARF type. 7](#_Toc185613391)

[Table S6. Echocardiography findings among ARF cases. 8](#_Toc185613392)

[Table S7. Number of ARF cases for which antibiotics were administered during admission, by antibiotic type and study site. 9](#_Toc185613393)

[Table S8. Steroid administration for ARF cases. 10](#_Toc185613394)

[Table S9. Length of stay according to ARF phenotype. 13](#_Toc185613395)

[Table 10. Crude ratio of length of stay: univariable regression. 14](#_Toc185613396)

[Table S11. Adjusted ratio of length of stay: final multivariable regression model. 16](#_Toc185613397)

[Figure S1. Venn diagram Venn diagram depicting clinical manifestations of ARF by Jones criteria feature. Carditis = valve features consistent with rheumatic changes on echocardiogram; Joint = polyarthralgia, polyarthritis or aseptic monoarthritis; EM = erythema marginatum; PR = prolonged PR; fever = subjective or objective (≥38·0°C) fever. 17](#_Toc185613398)

[Figure S2. Association between carditis and first-degree heart block (prolonged PR interval). Numbers in the bars are number of ARF cases. 18](#_Toc185613399)

# Table S1. START study data collected for analysis.

| - Age (years) - Gender (male or female) - Ethnicity (Aboriginal, Torres Strait Islander, Māori, Pacific Islander, and/or non-Indigenous) - Hospital site - Pregnancy (yes, no or unknown) - ARF type (definite, probable or possible^1^) - Recurrence (initial or recurrent) - Current or prior RHD diagnosis (yes or no) - Recency of RHD diagnosis (this admission, 0-2 years prior, >2 years prior) - Evidence of infection (i.e. sore throat, skin sores, antibiotics given for sore throat/skin sores; yes or no for each) - Time from symptom onset to admission (days) - Length of stay (days) - Findings on echocardiogram:   - mitral valve regurgitation (MVR) (none/physiologic, mild, moderate or severe)   - aortic valve regurgitation (AVR) (none/physiologic, mild, moderate or severe)   - mitral stenosis (yes or no)   - aortic stenosis (yes or no)   - tricuspid valve lesion (yes or no)   - pulmonary valve lesion (yes or no)   - pericardial effusion (yes or no)   - congenital health abnormality (yes or no)   - presence of clinical features as per the 2015 revised Jones criteria (high-risk settings), grouped into carditis (‘clinical’ and ‘subclinical’), joint involvement (aseptic monoarthritis, polyarthralgia and polyarthritis), skin involvement (erythema marginatum), fever, chorea and prolonged PR (yes or no)   - clinical combination (or ‘phenotypes’) as per the above groupings (for example, carditis + joint involvement) - Maximum recorded temperature (degrees Celsius) - Laboratory investigations:   - C-Reactive Protein (CRP) (maximum value)   - Erythrocyte Sedimentation Rate (ESR) (maximum value)   - Elevated CRP ≥30 mg/L (yes or no)   - Elevated ESR ≥30 mm/h (yes or no)   - White cell count (only one value available) (reference range 4-11 x 10^9^/L)   - Anti-streptolysin O (ASO) (maximum value)   - Anti-DNAse B (ADB) (maximum value)   - Culture source   - Culture result positive for Group A Strep (yes or no) - Medications:   - Antibiotics given (yes or no; name(s))   - Corticosteroids given (yes or no; name(s), dose and duration)   - Non-steroidal anti-inflammatory medication given (yes or no; name(s), dose and duration) |
| --- |

**Table S2. Laboratory results among ARF cases.**

| Feature | All | | Royal Darwin Hospital (AUS) | | Kidz First Hospital (NZ) | | Starship Hospital (NZ) | |  |
| --- | --- | --- | --- | --- | --- | --- | --- | --- | --- |
|  | N | n (%), median (IQR) | N | n (%), median (IQR) | N | n (%), median (IQR) | N | n (%), median (IQR) | p value (AUS vs NZ) |
| **Biochemical/serology results (n, %)** |  |  |  |  |  |  |  |  |  |
| Elevated CRP  (≥ 30 mg/L) | 141 | 103 (73%) | 87 | 60 (69%) | 33 | 27 (82%) | 21 | 16 (76%) | 0·2 |
| Elevated ESR  (≥ 30 mm/h) | 141 | 125 (89%) | 86 | 73 (85%) | 33 | 32 (97%) | 22 | 20 (91%) | 0·078 |
| Elevated ASO titre | 140 | 125 (89%) | 88 | 79 (91%) | 32 | 28 (85%) | 20 | 18 (90%) | 0·5 |
| Elevated ADB titre | 131 | 95 (73%) | 80 | 65 (81%) | 33 | 21 (64%) | 18 | 9 (50%) | **0·005** |
| **Biochemical/serology results (median, IQR)** |  |  |  |  |  |  |  |  |  |
| Highest CRP (mg/L) | 141 | 65·0 (25·3 to 107) | 87 | 56·7 (18·4 to 104) | 33 | 74·0 (58·0 to 113·0) | 21 | 71·0 (35·0 to 86·0) | 0·066 |
| Excluding chorea | 130 | 69·5 (35·2 to 108) | 79 | 59·1 (28·1 to 108) | 30 | 86·0 (63·5 to 121) | 21 | 71·0 (35·0 to 86·0) | 0·086 |
| Highest ESR (mm/h) | 141 | 82 (45 to 99) | 86 | 70·5 (35·3 to 91·5) | 33 | 94 (69 to 115) | 22 | 99 (60 to 128) | <**0·001** |
| Excluding chorea | 130 | 85 (53 to 100) | 78 | 76·5 (41·8 to 92·0) | 30 | 97 (71·5 to 118) | 22 | 99 (69·5 to 128) | <**0·001** |
| WBC (x 10^9^/L) | 142 | 9·90 (8·03 to 12·28) | 87 | 9·60 (7·80 to 12·25) | 33 | 11·4 (9·50 to 13·5) | 22 | 9·10 (7·98 to 11·53) | 0·13 |
| Peak ASO titre (U/ml) | 140 | 657 (411 to 988) | 87 | 652 (416 to 894) | 33 | 746 (438 to 1281) | 20 | 592 (404 to 948) | 0·3 |
| Highest ADB titre (units/ml) | 131 | 720 (420 to 925) | 80 | 825 (500 to 1125) | 33 | 720 (355 to 720) | 18 | 497 (225 to 720) | **<0·001** |
| **GAS Culture (n, %)** |  |  |  |  |  |  |  |  |  |
| Culture positive (any) | 120 | 39 (33%) | 67 | 1 (1%) | 33 | 29 (88%) | 20 | 9 (45%) | **<0·001** |
| Throat | 84 | 37 (44%) | 32 | 1 (3%) | 32 | 27 (84%) | 20 | 9 (45%) | **<0·001** |
| Skin sore | 4 | 1 (0%) | 2 | 0 | 1 | 1 (100%) | 1 | 0 | - |

ADB = anti-DNAse B, ARF = acute rheumatic fever, ASO = anti-streptolysin titre, AUS = Australia, CRP = C-reactive peptide, ESR = erythromycin sedimentation rate, GAS+ Group A Streptococcus; IQR = interquartile range, N = denominator, NZ = New Zealand, RHD = rheumatic heart disease, WBC = white blood cell count.

p-values were calculated using Pearson’s chi squared test, Fisher’s exact test or Wilcoxon rank sum test.

# Table S3. CRP and ESR by ARF type.

|  | CRP & ESR elevated | CRP elevated only | ESR elevated only | Neither elevated | Total tested | Total positive |
| --- | --- | --- | --- | --- | --- | --- |
| Definite ARF | 88 | 1 | 19 | 7 | 115 | 108/115 (94%) |
| Probable ARF | 9 | 0 | 6 | 0 | 15 | 15/15 (100%) |
| Possible ARF | 0 | 0 | 3 | 6 | 9 | 3/9 (33%) |
| (total) | 97 | 1 | 28 | 13 | 139 |  |

# Table S4. Preceding symptoms of a relevant infection and receipt of antibiotics

|  | **All** | | **Royal Darwin Hospital (AUS)** | | **Kidz First Hospital (NZ)** | | **Starship Children’s Hospital (NZ)** | | **p value (AUS vs NZ)** |
| --- | --- | --- | --- | --- | --- | --- | --- | --- | --- |
|  | **N** | **n (%)** | **N** | **n (%)** | **N** | **n (%)** | **N** | **n (%)** |  |
| Sore throat or skin sores in the 8 weeks prior to enrolment | 132 | 79 (60%) | 78 | 42 (54%) | 33 | 19 (58%) | 21 | 18 (86%) | **0·029** |
| Sore throat in the 8 weeks prior to enrolment | 132 | 55 (42%) | 80 | 26 (33%) | 32 | 14 (44%) | 20 | 15 (75%) | **0·003** |
| Skin sores in the 8 weeks prior to enrolment | 127 | 37 (29%) | 77 | 21 (27%) | 33 | 9 (27%) | 17 | 7 (41%) | **0·5** |
| Of those with symptoms, recalled receiving antibiotics* | 77 | 42 (55%) | 40 | 19 (48%) | 19 | 11 (58%) | 18 | 12 (67%) | 0·4 |
| Time from symptom onset to enrolment (days, median (IQR)) | 141 | 9 (5 to 20) | 86 | 8 (4 to 15) | 33 | 10 (7 to 17) | 22 | 19 (10 to 41) | **0·003** |

*Recall of antibiotics received for the preceding infection, not on suspicion of ARF or for treatment of ARF.

# Table S5. ASO and anti-DNaseB by ARF type.

|  | ASO & ADB elevated | ASO elevated only | ABD elevated only | Neither elevated | Total tested | Total positive |
| --- | --- | --- | --- | --- | --- | --- |
| Definite ARF | 75 | 23 | 5 | 4 | 107 | 103/107 (96%) |
| Probable ARF | 9 | 3 | 1 | 2 | 15 | 13/15 (87%) |
| Possible ARF | 5 | 2 | 0 | 1 | 8 | 7/8 (88%) |
| (total) | 89 | 28 | 6 | 7 | 130 |  |

# Table S6. Echocardiography findings among ARF cases.

| Feature | All | | Royal Darwin Hospital (AUS) | | Kidz First Hospital (NZ) | | Starship Hospital (NZ) | |
| --- | --- | --- | --- | --- | --- | --- | --- | --- |
|  | N | n (%), | N | n (%), | N | n (%), | N | n (%), |
| Normal echo | 143 | 56 (39%) | 88 | 35 (40%) | 33 | 12 (36%) | 22 | 9 (41%) |
| Mitral regurgitation (mild/moderate/ severe) | 143 | 78 (55%) | 88 | 48 (55%) | 33 | 18 (55%) | 22 | 12 (55%) |
| Mild | 143 | 46 (32%) | 88 | 28 (32%) | 33 | 12 (36%) | 22 | 6 (27%) |
| Moderate | 143 | 19 (13%) | 88 | 14 (16%) | 33 | 4 (12%) | 22 | 1 (5%) |
| Severe | 143 | 13 (9%) | 88 | 6 (7%) | 33 | 2 (6%) | 22 | 5 (23%) |
| Aortic regurgitation (mild/moderate/ severe) | 143 | 41 (29%) | 88 | 21 (24%) | 33 | 11 (33%) | 22 | 9 (41%) |
| Mild | 143 | 30 (21%) | 88 | 17 (19%) | 33 | 8 (24%) | 22 | 5 (23%) |
| Moderate | 143 | 6 (4%) | 88 | 3 (3%) | 33 | 1 (3%) | 22 | 2 (9%) |
| Severe | 143 | 5 (3%) | 88 | 1 (1%) | 33 | 2 (6%) | 22 | 2 (9%) |
| Mitral and aortic valve abnormalities | 143 | 32 (22%) | 88 | 16 (18%) | 33 | 8 (24%) | 22 | 8 (36%) |
| Mitral stenosis | 143 | 9 (6%) | 88 | 6 (7%) | 33 | 1 (3%) | 22 | 2 (9%) |
| Pericardial effusion* | 138 | 7 (5%) | 88 | 2 (2%) | 33 | 2 (6%) | 22 | 3 (14%) |
| Congenital heart abnormality | 143 | 1 (1%) | 88 | 0 | 33 | 1 (3% | 22 | 0 |

AUS = Australia, N = denominator, NZ = New Zealand.

*One large pericardial effusion with tamponade in a child with severe mixed valvular disease; other effusions were small/trivial.

# Table S7. Number of ARF cases for which antibiotics were administered during admission, by antibiotic type and study site.

| Antibiotic class | All  (n = 139) | Royal Darwin Hospital  (n = 86) | Kidz First Hospital  (n = 33) | Starship Hospital  (n = 20) |
| --- | --- | --- | --- | --- |
| Any antibiotic | 125 | 76 | 31 | 18 |
| Penicillins* | 100 | 72 | 13 | 15 |
| Other penicillins (ampicillin, amoxicillin, amoxicillin/clavulanic acid, flucloxacillin, piperacillin/tazobactam) | 38 | 9 | 21 | 8 |
| Cephalosporins (cefalexin, ceftriaxone, cephazolin) | 11 | 8 | 1 | 2 |
| Sulfamethoxazole/trimethoprim | 4 | 4 | 0 | 0 |
| Aminoglycosides (gentamicin) | 2 | 2 | 0 | 0 |
| Glycopeptides (vancomycin) | 1 | 1 | 0 | 0 |
| Metronidazole | 1 | 1 | 0 | 0 |
| Tetracyclines (doxycycline) | 1 | 1 | 0 | 0 |

Note: Participants could be prescribed more than one antibiotic.

*Penicillins were variously recorded as any of the following: penicillin, intravenous penicillin, intramuscular penicillin, oral penicillin, penicillin V, benzathine penicillin, benzathine benzylpenicillin, benzylpenicillin, phenoxypenicillin.

# Table S8. Steroid administration for ARF cases.

| Case | Age | Weight | Steroid/s | Indication | Dosage, duration, weaning plan |
| --- | --- | --- | --- | --- | --- |
| 1 | Pre adolescence | 23kg | prednisolone | Carditis with severe AR | 1mg/kg daily for 7 days  2mg/kg daily for 6 days (due to worsening pulse pressure)  Discharged on 2mg/kg daily to interstate hospital for cardiac surgery  Documented weaning plan for after surgery:  1mg/kg daily for 7 days  0.5mg/kg daily for 7 days  0.25mg/kg daily for 7 days  Cease  Total duration on prednisolone: 13 days prior to interstate transfer, unknown duration after this |
| 2 | Pre adolescence | 29.8kg | prednisolone | Severe MR | Commenced prednisolone on day 2.  2mg/kg (55mg) daily, 6 doses over 7 days  1.5mg/kg (40mg) daily for 7 days  1mg/kg (25mg) daily for 7 days  Ceased  Discharged to HitH on day 23  Re-admitted to the ward and prednisolone re-started on day 30:  2mg/kg (60mg) daily for 4 days  1mg/kg (30mg) daily for 11 days  Discharged to HitH on day 44. Weaning plan for HitH:  1mg/kg (30mg) daily for 7 days  0.5mg/kg (15mg) daily for 7 days  Discharged from HitH on day 62. Weaning was intended to continue with 0.3mg/kg (10mg) daily for 7 days, then cease, but clinical records suggest that this did not occur.  Total estimated duration on prednisolone: 49 days |
| 3 | Pre adolescence | 24.4kg | prednisolone | ARF with moderate MR | Commenced prednisolone on day 3.  1mg/kg (20mg) daily for 24 days  0.5mg/kg (10mg) daily for 5 days  Discharged to HitH  Weaning doses given in HitH:  0.25mg/kg (5mg) daily for 5 days  0.125mg/kg (2.5mg) daily for 5 days  Ceased  Total duration on prednisolone: 39 days |
| 4 | Mid adolescence | Approx. 40kg | prednisolone | ARF with heart failure, severe MR and mild AR | Commenced prednisolone on day 2.  1mg/kg (40mg) daily for 11 days  0.5mg/kg (20mg) daily for 6 days  1mg/kg (40mg) daily for 11 days (rebound inflammatory markers, ?worsening carditis)  Discharged to HitH on day 30. Discharged from HitH on day 57.  Weaning plan for HitH admission:  0.5mg/kg (20mg) daily for 14 days  0.25mg/kg (10mg) daily for 14 days  0.125mg/kg (5mg) daily for 14 days  Cease  Total intended duration of prednisolone: 72 days |
| 5 | Mid adolescence | 64.5kg | Methylprednisolone,  prednisolone | Possible small vessel vasculitis, Sydenham’s chorea | Methylprednisolone commenced on day 6.  1.3mg/kg (1g) for 3 days (possible small vessel vasculitis)  No steroid day 9  Prednisolone commenced on day 10.  2mg/kg (80mg) daily for 3 days  Discharged on day 13.  As per discharge summary: weaning plan was 2mg/kg (80mg) daily to complete 7 days  1.5mg/kg (approx. 60mg) daily for 7 days  1mg/kg (approx. 40mg) daily for 7 days  50mg for 5 days  25mg for 5 days  Last available clinical record indicates that weaning continued at 20mg, unknown duration and unknown further weaning  Total known duration on corticosteroids: at least 34 days |
| 6 | Mid adolescence | 96kg | methylprednisolone | Given in PICU for heart block | Methylprednisolone commenced on day 1.  1g daily, 3 doses over 3 days.  Ceased  Total duration on methylprednisolone: 3 days |
| 7 | Mid adolescence | 32.5kg | prednisolone | Carditis with acute rheumatic valvulitis, moderate-to-severe MR | Prednisolone commenced on day 3.  1mg/kg (37.5mg) daily for 10 days  0.5mg/kg (20mg) daily for 1 day  0.25mg/kg (10mg) daily for 1 day  0.125mg/kg (5mg) daily for 1 day  Ceased, no doses for 3 days but then recommenced due to worsening inflammatory markers:  0.5mg/kg (16mg) daily for 4 days  0.25mg/kg (10mg) daily for 1 day  0.125mg/kg (5mg) daily for 1 day  Ceased before discharge  Total duration on prednisolone: 19 days |

HitH = Hospital in the Home. This is a service provided to children who are well enough to be discharged from the hospital ward, but whose living situation (too remote, unable to make regular visits to specialist clinics) is not suitable for being managed in an outpatient clinic.

# Table S9. Length of stay according to ARF phenotype.

| **ARF phenotype** | **N** | **Length of stay in days, median (range)** |
| --- | --- | --- |
| Carditis, Joint involvement, Fever, Prolonged PR, Chorea | 1 | 46 (46 to 46) |
| Carditis, Joint involvement, Erythema marginatum, Fever, Prolonged PR | 2 | 34 (2 to 66) |
| Carditis, Chorea | 3 | 26 (9 to 49) |
| Carditis, Fever, Prolonged PR | 4 | 20 (9 to 46) |
| Carditis, Joint involvement, Fever | 12 | 14 (3 to 31) |
| Carditis, Joint involvement, Erythema marginatum, Prolonged PR | 1 | 14 (14 to 14) |
| Carditis, Joint involvement, Prolonged PR | 16 | 12 (3 to 46) |
| Carditis, Joint involvement, Chorea | 1 | 12 (12 to 12) |
| Carditis | 3 | 11 (11 to 14) |
| Carditis, Joint involvement | 12 | 10 (2 to 29) |
| Joint involvement, Erythema marginatum, Fever, Prolonged PR | 2 | 8 (8 to 8) |
| Carditis, Joint involvement, Erythema marginatum | 2 | 8 (4 to 11) |
| Carditis, Joint involvement, Fever, Prolonged PR | 27 | 8 (2 to 38) |
| Carditis, Prolonged PR, Chorea | 2 | 7 (7 to 7) |
| Joint involvement, Prolonged PR | 12 | 7 (3 to 16) |
| Joint involvement, Fever | 14 | 7 (2 to 15) |
| Joint involvement, Chorea | 1 | 6 (6 to 6) |
| Chorea | 2 | 6 (2 to 9) |
| Joint involvement, Fever, Prolonged PR | 15 | 5 (2 to 14) |
| Carditis, Fever, Chorea | 1 | 4 (4 to 4) |
| Joint involvement | 9 | 4 (2 to 8) |
| Erythema marginatum | 1 | 3 (3 to 3) |

# Table S10. Crude ratio of length of stay: univariable regression.

| **Characteristic** | **N** | **Length of Stay OR** | **95% CI** | **p-value** |
| --- | --- | --- | --- | --- |
| Country | 141 |  |  |  |
| Australia |  | — | — |  |
| New Zealand |  | 1.59 | 1.24, 2.05 | **<0.001** |
| Study site | 141 |  |  |  |
| Royal Darwin Hospital |  | — | — |  |
| Kidz First Hospital, Auckland |  | 1.34 | 1.00, 1.80 | 0.05 |
| Starship Children’s Hospital, Auckland |  | 2.12 | 1.49, 3.02 | **<0.001** |
| Age | 141 | 1.01 | 0.97, 1.05 | 0.5 |
| Gender | 141 |  |  |  |
| Female |  | — | — |  |
| Male |  | 0.98 | 0.75, 1.28 | 0.9 |
| ARF certainty (Definite vs Probable/Possible) | 141 | 1.48 | 1.18, 1.87 | **0.001** |
| ARF episode | 141 |  |  |  |
| Initial |  | — | — |  |
| Recurrent |  | 0.92 | 0.64, 1.34 | 0.7 |
| Time between symptom onset & enrolment, per 5 days | 139 | 1.08 | 1.03, 1.13 | **0.001** |
| Presence of preceding symptoms |  | 1.24 | 0.95, 1.62 | 0.11 |
| Peak ESR, per 50 mm/hr | 139 | 1.64 | 1.38, 1.94 | **<0.001** |
| Peak CRP, per 50 mg/L | 140 | 1.18 | 1.05, 1.34 | **0.008** |
| Peak ASO, per 500 units/ml | 138 | 1.13 | 1.00, 1.29 | **0.049** |
| Peak ADB, per 500 units/ml | 129 | 0.94 | 0.83, 1.05 | 0.3 |
| Positive culture (any) | 141 | 1.26 | 0.94, 1.68 | 0.12 |
| Carditis | 141 | 1.89 | 1.49, 2.40 | **<0.001** |
| Chorea | 141 | 1.22 | 0.76, 1.96 | 0.4 |
| Erythema marginatum | 141 | 0.92 | 0.53, 1.60 | 0.8 |
| Joint involvement | 141 | 0.77 | 0.52, 1.15 | 0.2 |
| Prolonged PR | 141 | 1.18 | 0.92, 1.53 | 0.2 |
| Fever | 141 | 1.11 | 0.86, 1.43 | 0.4 |
| Presence of mitral/aortic valve disease | 141 | 1.89 | 1.49, 2.40 | **<0.001** |
| Given steroids | 141 | 2.48 | 1.41, 4.38 | **0.002** |

# Table S11. Adjusted ratio of length of stay: final multivariable regression model.

| **Characteristic** | **Length of stay OR** | **95% CI** | **p-value** |
| --- | --- | --- | --- |
| Country |  |  |  |
| AUS | — | — |  |
| NZ | 1·25 | 0·99, 1·59 | 0·068 |
| Definite ARF (versus Probable/Possible ARF) | 1·02 | 0·81, 1·27 | 0·9 |
| Time from symptom onset to enrolment, per 5 days | 1·04 | 0·99, 1·08 | 0·1 |
| Peak ESR, per 50mm/hr | 1·4 | 1·17, 1·67 | <0·001 |
| Presence of mitral/aortic valve disease | 1·56 | 1·23, 1·98 | <0·001 |
| Given steroids | 1·99 | 1·22, 3·26 | 0·007 |

ARF = acute rheumatic fever, AUS = Australia, CI = confidence interval, ESR = erythrocyte sedimentation rate, NZ = New Zealand, OR = odds ratio.

# Figure S1. Venn diagram Venn diagram depicting clinical manifestations of ARF by Jones criteria feature. Carditis = valve features consistent with rheumatic changes on echocardiogram; Joint = polyarthralgia, polyarthritis or aseptic monoarthritis; EM = erythema marginatum; PR = prolonged PR; fever = subjective or objective (≥38·0°C) fever.


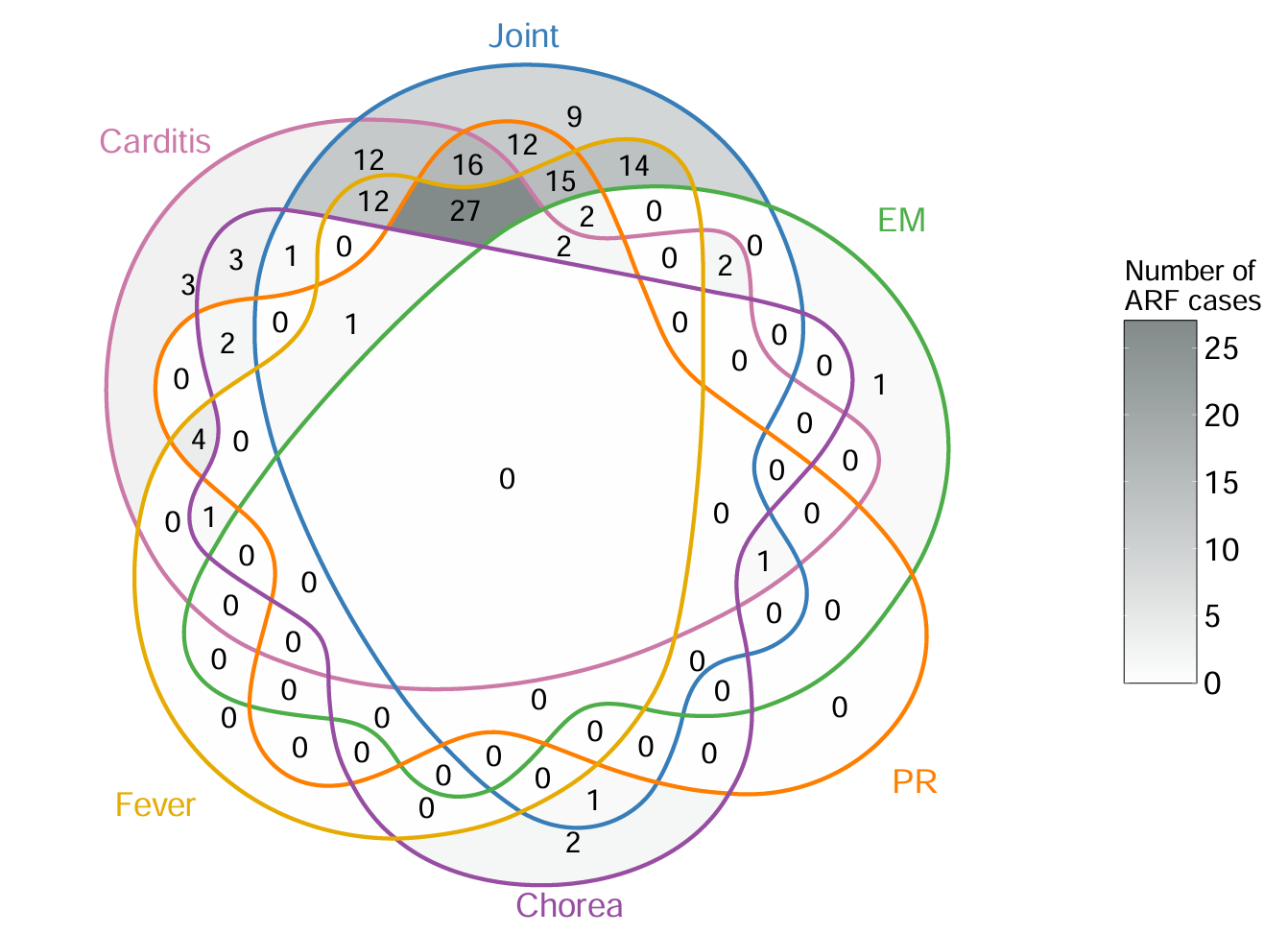


# Figure S2. Association between carditis and first-degree heart block (prolonged PR interval). Numbers in the bars are number of ARF cases.
