# Supplementary material for: Diverse diagnostic and management approaches for acute rheumatic fever in Australia and New Zealand: findings of a prospective clinical study
Source: BMJ Open. 2025 Jul 17;15(7):e099827. doi: 10.1136/bmjopen-2025-099827 (PMC12273072; doi:10.1136/bmjopen-2025-099827)
Supplement: Supplementary file 2 [file bmjopen-2025-099827.reviewer_comments.pdf]

PEER REVIEW HISTORY

BMJ Open publishes all reviews undertaken for accepted manuscripts. Reviewers are asked to complete a checklist review form and are provided with free text boxes to elaborate on their assessment. These free text comments are reproduced below.

ARTICLE DETAILS

Title (Provisional)

Diverse diagnostic and management approaches for acute rheumatic fever in Australia and New Zealand: findings of a prospective clinical study

Authors

Peiris, Ruwani; Webb, Rachel; Bennett, Julie; Yan, Jennifer; Francis, Joshua Reginald; Remenyi, Bo; Chan Mow, Florina; Burgess, Rachel; Wilson, Nigel; Stanley, Alicia; Francis, Laura; Holloway, Rhonda; Westbury, Roxanne; Lawrence, Shirley; Hernandez-Gomez, Yolanda; Broadhurst, David; Moreland, Nicole J; McGregor, Reuben; Motteram, Caroline; Pearson, Glenn; Mayo, Mark; Ralph, A; Carapetis, Jonathan

---

VERSION 1 - REVIEW

**Reviewer**	**1**
**Name**	**Marino, Achille**
**Affiliation**	**Gaetano Pini-CTO**
**Date**	**16-Mar-2025**
**COI**	**None**

This study examines differences in the diagnosis and management of acute rheumatic fever (ARF) in Australia and New Zealand, using a prospective multicenter study design (2018–2021). Clinical presentation, hospitalization duration, and therapeutic approaches were compared across three hospitals (1 AUS, 2 NZ). Its key findings could be considered the difference in international clinical practice and the need for standardization in ARF management.

The topic of the study is significant, particularly considering the high rate of ARF in these countries. Overall, the manuscript is well-written. Nevertheless, the authors spend too much time on the descriptive parts, often overlapping results with table data. This leads to long descriptive results that are hard to follow. Despite the description of differences between AU and NZ management of ARF, the paper's focus, as stated in the methods, is to find factors associated with length of hospitalization; this part should be widened in the results and adequately discussed inthe relative paragraph. Other caveats are the differences in

healthcare access and ethnic variability within the cohorts. Indeed, the ethnic variability of presentations and outcomes would be a interesting area to expand.

Here are my suggestions:

- Abstract: add numbers/rates instead of using sentences like “Positive throat Group A Streptococcus culture was more common in New Zealand”

- 89-92: I would delete this, since is matter already known! I'd rather stress the factor associated with the length of stays and the differences in management found.

- 118-122: I do not understand, is NZ using the guideline for high-risk populations? Please clarify.

- 123-124: I suggest explicating possible/probable ARF according to AU and NZ

- 129: “healthy” controls

- 131: replace “diagnostic approaches” with “clinical an laboratory features” since you described the latter without analyzing the diagnostic processes.

- 246-249: standard deviation should be used to compare BMI.

- The following paragraphs duplicate table 1, table 2, or table 3: ARF certainty and ARF recurrence, Rheumatic heart disease, Clinical manifestations of ARF, Treatments received. I suggest reducing them and keeping just data not shown on tables. Particularly, I would keep the description of patients with ARD recurrences, adding information on secondary prophylaxis and the outcome.

- 293: WCC? Is it WBC? Please clarify

- 307: how many patients in your cohort who developed ARF have a history of skin sores?

- 376: why the rate of NSAID users is so low?

- 392: Hospital length of stay and associated factors

- 410-417: please expand this, reporting Ors values, Cis, and p of what you consider significant (clinically significant). Be aware of the possible risk of collinearity between corticosteroid use and valve disease in the multivariable analysis (use the appropriate test to assess it).

- 422-423: your study did not report differences in measures of illness severity (eventually in the carditis severity), streptococcal infection epidemiology (not at all), diagnostic practices (herein you described clinical and laboratory features along with received treatments), especially in relation to the classification of RHD (this wasn't deepened, look at lines 339-341) in the acute setting, and variations in management approaches.

- 455-461: move it to line 506

- 514: why low rates of NSAIDs users?

- 537-542: The variation over time of ASO is more “diagnostic” of a close GAS infection than the single value. Did you capture this variation in the study? If not, please state it as a limitation.

- Please add a comment on factors associated with the length of hospitalization and future possible strategies to reduce it.

- Please expand the study limitations in light of these considerations.

**Reviewer**	**2**
**Name**	**Mutagaywa, Reuben**
**Affiliation**	**Muhimbili University of Health and Allied Sciences**
**Date**	**23-Mar-2025**
**COI**	**None**

Thank you for calling me to review this article.
I read the article with great interest.
Acute rheumatic fever (ARF) remains a worldwide cause of heart failure, stroke and maternal mortality. However, there is a lack of a known ARF diagnostic testing owing to several reasons like poor health-seeking behavior, varied disease presentations, overlapping with other febrile illnesses such as malaria, and an incomplete understanding of disease pathogenesis. Moreover, the management of ARF including the use of secondary antibiotic prophylaxis is varying from region to region of disease endemic areas signifying lack of understanding of this disease. To address these barriers, studies like the current one are needed.

Hereunder, are my comments

MANUSCRIPT REVIEW:

Study title: Diverse diagnostic and management approaches for acute rheumatic fever in Australia and New Zealand: findings of a prospective clinical study

Manuscript Number: bmjopen-2025-099827

Article Type: Original research

Authors: Ruwani Peiris et al

Corresponding Author: Anna P. Ralph

General Comment: A very important study of public health importance. Findings from this study will be eye opener for best clinical practice for management of patients with ARF. Authors are sharing initial data from START Study.

Abstract

Introduction: Focused to the study

Methods: Could briefly mention what are those clinical data

Results: The author conclude that significant predictor for length of stay were corticosteroids and higher ESR – are these two variables not linearly correlated?

Conclusion: I don't think this study adds new findings from what is known for decades. May be if it is a proof of concept. For example, ESR has been in the original (1944) Jones criteria, modified/revised criteria of 2018, 2020, and 2018. The same applies to application of AntiDNase B. Can authors argue on this?

1.1 Background

Short and focused to the study

1.2 Problem statement and rationale

Mentioned

1.3 Study objectives

Clearly outlined

1.4 Research methods

Study design: Appropriate

i) How was the study sites chosen? …authors mention that "these hospitals service regions with the highest rates of ARF in their respective countries" …are they representative of rest study population? …Any sampling procedure done?

ii) The description of intervention is explicitly mentioned

iii) Inclusion criteria: Why differing age group of inclusion per study sites? i.e Royal Darwin Hospital (aged 5 to 30 years) vs Starship Hospital (aged 2 to 20 years). For Kidz First the included participants by age was not mentioned.

iv) Diagnostic process: The study intended to recruit participants up to age of 30yrs (eventually the oldest individual was 23 years) but there was no adult cardiologist involved in the panel to review case record forms and primary data.

v) Ethical clearance: **Mentioned**

1.5 Results

Nicely presented

1.6 Discussion

Authors report that "Useful information for people diagnosed with ARF and their families include details …". Although it sound to be important information but it is not supported by the findings from the current study i.e was not researched on!

1.7 Conclusion

Authors argue that the study provided new information. As I previously commented, most of the information are not new. May be the author could say that their findings are proof of concept of what has been known from the past.

1.8 Study limitations

Were clearly put forward

1.9 Dissemination of findings

Well planned

1.10 Conflict of interest

Authors declare no conflict of interest

1.11 Originality of Contribution

Relatively similar work has been done in the past.

1.12 Literature Citation

Good

1.13 Final Evaluation/Recommendation for Publication

Is worthy publication after responding to above comments

1.14 References

These are up-to-date citations

**Reviewer**	**3**
**Name**	**Shimanda, Panduleni Penipawa**
**Affiliation**	**Department of Epidemiology and Global Health, Umeå University**
**Date**	**26-Mar-2025**
**COI**	**None**

Abstract: key words are missing

Page 11, line 254: '118/143' should be episodes not participants. Participants were 141

Page 11, line 258: Clarify whether those are participants or episodes. The entire table 1 presents episodes and not total number of participants.

Page 11, line 261-266: Clarify whether 'cases' refers to number of patients or episodes.

Page 15, line 338-350: Clarify the presentation of RHD whether it is on number of patients or per ARF episodes. Table 1 presented only the number of episodes. Also, the total of 44 differs from 41 in table 1 and description at page 11.

Page 16, line 349: Clarify whether the three participants are not included in the 41 as presented in table 1. This could be the difference between text on page 15 and page 11.

VERSION 1 - AUTHOR RESPONSE

	Reviewer: 1	
1	…Overall, the manuscript is well-written. Nevertheless, the authors spend too much time on the descriptive parts, often overlapping results with table data. This leads to long descriptive results that are hard to follow. Despite the description of differences between AU and NZ management of ARF, the paper's focus, as stated in the methods, is to	We are grateful for the feedback. The Results section has now been trimmed significantly in a number of places. This has resulted in the word count staying <5000 despite extensive additions requested below (e.g. definitions of

	find factors associated with length of hospitalization; this part should be widened in the results and adequately discussed in the relative paragraph. Other caveats are the differences in healthcare access and ethnic variability within the cohorts. Indeed, the ethnic variability of presentations and outcomes would be a interesting area to expand.	probable and possible ARF adds 112 words; inclusion of ORs and 95% CIs in the text as well as tables adds 100 words).<br>We have added to the discussion on length of hospitalisation as requested, and note that this is both a marker of disease severity, and clinical preferences, noting differences in practice at different sites.<br>The differences in ethnic variability of presentations were few; the differences that were evident are highlighted in the results. Our data do not explore healthcare access issues; we provide commentary in the discussion relating to possible consequences of differential healthcare access in relation to secondary prophylaxis and differences in ARF recurrence rates.
2	- Abstract: add numbers/rates instead of using sentences like "Positive throat Group A Streptococcus culture was more common in New Zealand"	We have added numbers to the sentences in 64-67:<br>"Australian participants more commonly had ARF recurrence (22% vs 0%), RHD (48% vs 0%), possible/probable ARF (23% vs 9%) and underweight (64% vs 16%). No instances of RHD were diagnosed among participants in New Zealand. Positive throat Group A Streptococcus culture was more common in New Zealand (69% vs 3%)."
3	- 89-92: I would delete this, since is matter already known! I'd rather stress the factor associated with the length of stays and the differences in management found.	We would like to retain this finding, as the added value of ESR over CRP alone, and anti-DNase B over ASO alone, in meeting diagnostic criteria for ARF, was a strong topic of debate among clinicians while this study was being conducted. We believe that our findings quantify the value of this testing for clinicians (not previously reported), despite the challenges accessing these tests.
4	- 118-122: I do not understand, is NZ using the guideline for high-risk populations? Please clarify.	NZ uses different ESR and CRP cutoffs for high risk populations from the Jones Criteria and Australian Criteria for high risk populations. Additionally, NZ does not consider polyarthralgia a major

		criterion. This is to improve specificity of the high-risk criteria in the NZ context.<br><br>We have modified this section to provide more clarity in the manuscript:<br>"there are different criteria for high and low risk settings, and international variations. For example, New Zealand applies the high-risk criteria but with modifications for improved specificity: polyarthralgia is a minor (not major) criterion, monoarthritis is not included, and higher cut-offs are applied for erythrocyte sedimentation rate (ESR; ≥50 instead of ≥30mm/hr), C-reactive protein (CRP; ≥50 instead of ≥30mg/L), and streptococcal serology.[1,2] To avoid missing subtle cases, 'probable' and 'possible' ARF diagnoses are defined in the Australian and New Zealand guidelines.[1,3] Probable (highly suspected) ARF is defined as an "acute presentation which does not fulfil Jones diagnostic criteria for ARF, missing one major or one minor criterion or lacking evidence of preceding streptococcal infection, but ARF is still considered the most likely diagnosis," and possible (uncertain) ARF as the same, where "ARF is considered uncertain but cannot be ruled out".[1,2] AHA by contrast includes only 'possible ARF' as a clinical presentation where there is good reason to suspect ARF but criteria are not met due to missing results, limited documentation or unclear clinical features"
5	- 123-124: I suggest explicating possible/probable ARF according to AU and NZ	We have done so in the revised third paragraph replicated above.
6	- 129: "healthy" controls	There were not only healthy controls, there were 4 different control groups.<br>We now say:<br>"ARF compared with control diagnoses ("healthy", non-ARF Streptococcal infections, other acute conditions and RHD)."

7	- 131: replace “diagnostic approaches” with “clinical and laboratory features” since you described the latter without analyzing the diagnostic processes.	We feel that it is appropriate to use the wording ‘diagnostic approaches’. There is no diagnostic test for ARF, and this paper highlights the subjectivity inherent in ARF diagnostic practices: the Jones criteria are subject to differing interpretation, and we found unexpectedly that the interpretation of echocardiograms (especially the conundrum of acute vs acute on chronic changes) differed greatly between sites, resulting in patients receiving different diagnoses, depending on where they were enrolled into the study. Half the patients in Australia were diagnosed with RHD and none in NZ. The different diagnostic approach underpinning that outcome is described in the Discussion. Diagnostic approaches using a clinical review panel are covered in the Methods, and the different diagnostic approaches are detailed in the Results and Discussion.
8	- 246-249: standard deviation should be used to compare BMI.	The measure of distribution of BMI is presented as interquartile range in Table 1. This is not replicated in the text, in keeping with reviewer comment #1 to avoid presenting overlapping information in text and tables.
9	- The following paragraphs duplicate table 1, table 2, or table 3: ARF certainty and ARF recurrence, Rheumatic heart disease, Clinical manifestations of ARF, Treatments received. I suggest reducing them and keeping just data not shown on tables.	We have now condensed these paragraphs, including removing data from the text but retaining words as requested here and in comment #1. We believe that some interpretative text is required since understanding is otherwise lost. Comment #7 highlights that some level of explication is needed beyond that provided by tables alone.
10	I would keep the description of patients with ARF recurrences, adding information on secondary prophylaxis and the outcome.	We include all the available information available on antibiotic treatment (the first dose of which provides the initiation of secondary prophylaxis).<br>This is not intended to be an outcomes paper; it is about approaches to clinical diagnosis, and does not include follow up.

11	- 293: WCC? Is it WBC? Please clarify	Yes, WCC = white cell count (S Table 2 footnote). This has been changed to white blood cell count in the text and Supplementary Table 2.
12	- 307: how many patients in your cohort who developed ARF have a history of skin sores?	This is in Supplementary Table 4: of 127 participants with adequate data, 37 (29%) said they recalled having had skin sores in the 8 weeks prior to enrolment in the study.
13	- 376: why the rate of NSAID users is so low?	We make reference to this is line 526-528: "While some patients with mild or quickly resolving joint pain may not need NSAIDs, it is important to ensure that all patients are receiving adequate pain relief and that ARF management follows best practice."<br><br>We did not investigate reasons for the NSAID prescription rate, and so we have not commented further on this.
14	- 392: Hospital length of stay and associated factors	We have amended the heading to read "Hospital length of stay and associated factors".
15	- 410-417: please expand this, reporting Ors values, Cis, and p of what you consider significant (clinically significant). Be aware of the possible risk of collinearity between corticosteroid use and valve disease in the multivariable analysis (use the appropriate test to assess it).	OR, 95% CI and p values now added throughout the paragraph. This duplicates what is already presented elsewhere, but we appreciate that not all readers will review the supplementary materials.
16	- 422-423: your study did not report differences in measures of illness severity (eventually in the carditis severity), streptococcal infection epidemiology (not at all), diagnostic practices (herein you described clinical and laboratory features along with received treatments), especially in relation to the classification of RHD (this wasn't deepened, look at lines 339-341) in the acute setting, and variations in management approaches.	Illness severity is captured in many of the descriptors presented including ARF certainty (definite versus probable and possible), inflammatory marker levels, degree of valvular involvement, and patient and family impact as reflected in hospital length of stay. Streptococcal infection epidemiology is addressed clinically (self reported skin sores and sore throats), microbiologically and serologically. Significant differences are reported in streptococcal epidemiology in the patient groups from the different study settings as evident in the higher rates of reported sore throat (Supp Table 4) and culture positivity from throats (Table 1) in NZ vs Australia.

		Diagnostic practices are addressed in response to comment #7.<br>Management approaches refer to treatment received, and duration of hospitalisation, covered in the results, tables and discussion. The extent to which length of stay is a product of different management practices versus illness severity is described in detail in the third paragraph of the discussion.
17	- 455-461: move it to line 506	The lines immediately before 455-461 (now numbered differently due to edits) refer to practice differences between sites in relation to hospitalisation. Therefore, it seems most logical for the section in 455-461, which is also about practice differences between the sites (in relation to antibiotic timing and administration), to stay where it is.
18	- 514: why low rates of NSAIDs users?	Discussed above.
19	- 537-542: The variation over time of ASO is more "diagnostic" of a close GAS infection than the single value. Did you capture this variation in the study? If not, please state it as a limitation.	In clinical practice at both sites, multiple blood draws from children are rarely available. We have amended the following sentence to include streptococcal serology: "Limitations include the variable enrolment time in relation to ARF onset date, given variation in measures that evolve quickly such as symptoms, inflammatory marker concentration, streptococcal serological titres and ECG changes."
20	- Please add a comment on factors associated with the length of hospitalization and future possible strategies to reduce it.	We agree that duration of hospitalisation should be minimised if possible, but note that guidelines strongly recommend hospitalisation for all cases of ARF, and the rebound phenomenon which occurs on premature weaning of anti-inflammatory medications means that many cases inherently require prolonged hospital stays (especially when place of residence is hundreds of kilometres away from a hospital).<br><br>In this section, we note: 'Hospital length of stay was greatest if significant carditis

21		was present. Moderate or severe carditis occurring with other major criteria was often associated with particularly long hospitalisation requirements of a month or more…' We present the factors associated with length of stay in the final paragraph of the results, Supplementary Table 10 and Supplementary Table 11. We also note that 'Prolonged bed rest for rheumatic carditis had been a recognised practice among some practitioners at Starship Children's Hospital until recently, and the legacy of that practice remains evident.'<br>We have added the sentence: 'Revised editions of national ARF management guidelines are being published in both New Zealand and Australia in 2025, which respectively recommend early discharge for clinically stable patients and potential hospital avoidance in some selected cases.'
21	- Please expand the study limitations in light of these considerations.	We have expanded the limitations section to note time effects on streptococcal serology titres, and to note that this was not a longitudinal follow up study in which we can present outcomes (comments #1 and #10).
	Reviewer: 2	
1	General Comment: A very important study of public health importance. Findings from this study will be eye opener for best clinical practice for management of patients with ARF. Authors are sharing initial data from START Study.	-
2	Abstract Methods: Could briefly mention what are those clinical data	We are constrained by the word limit to list all the clinical data (symptoms, medical history, in-hospital treatment) but for better representation in the abstract of what was analysed, we have amended this sentence to "We analysed clinical, biochemical and echocardiographic data from a multi-site, prospective cohort ..."
3	Results: The author conclude that significant predictor for length of stay were corticosteroids and higher ESR – are these two variables not linearly correlated?	No. There was slightly higher ESR in the group given corticosteroids, this was not significantly different and there was substantial overlap.

		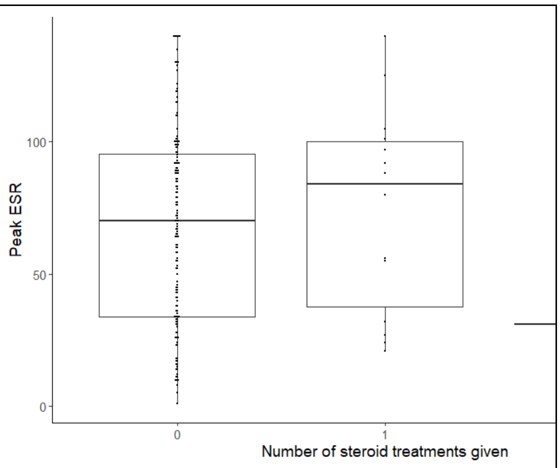<br>This is supported by the findings of Licciardi et al who noted that ESR and a higher degree of carditis were not positively correlated (https://doi.org/10.1007/s00246-024-03547-0).
4	Conclusion: I don't think this study adds new findings from what is known for decades. May be if it is a proof of concept. For example, ESR has been in the original (1944) Jones criteria, modified/revised criteria of 2018, 2020, and 2018. The same applies to application of AntiDNase B. Can authors argue on this?	New findings include but are not limited to:<br>- that diagnostic criteria including interpretation of echocardiographic findings in ARF are applied differently in different international settings; this needs to be resolved to promote further progress in multi-site diagnostic and treatment studies<br>- The percentage incremental yield of ESR over CRP (21%) has not previously been reported, which is why clinicians have lacked guidance on the need to do both, when ESR is inaccessible<br>- the percentage incremental yield of ABD over ASO (5%) has also not previously been reported, yet some local labs have discontinued access to ABD and clinicians have not been aware of the small incremental value of including this test, if ASO is negative<br>- the proportion of carditis cases with first degree heart block (61%, not significantly different from the proportion without carditis) has not previously been reported<br>- major differences in rates of detection of streptococcus in throat samples was

		identified from study sites, suggesting differing epidemiology at these sites<br>- the phenotypic similarities and differences in ARF presentations across study sites including proportion of all cases that present as chorea, or with subcutaneous nodules etc; proportion of cases that are definite, probable or possible; proportion of cases that are initial or recurrent<br>- that many Australian children with ARF are malnourished, while many New Zealand children with ARF are overweight or obese<br>- that hospital length of stay for ARF can be predicted by ESR, carditis and requirement for steroid treatment.<br><br>We have added wording to highlight the percentage incremental yield of ESR over CRP, which has not previously been described. ESR is an inaccessible, difficult test. It cannot be done in remote settings in Australia as described in the manuscript. Therefore its inclusion in local diagnostic guidelines has been questioned. Our findings indicate that getting access to an ESR test may result in a child being diagnosed with ARF when previously they might not have met diagnostic criteria. However, if the CRP has already been done and is above the cut-off for ARF, no ESR is required.<br><br>Similarly, the extent to which antiDNaseB adds to positive ARF case detection on top of ASO has not previously been clearly reported. We have added wording on the %incremental yield. This is relevant since many labs do not offer antiDNaseB testing, and advocacy is required to provide this test. Follow-up venepuncture with convalescent serology is rarely available, and there is a need for point of care data to facilitate

		decision making in the community prior to transfer to hospital.
5	1.1 Background: Short and focused to the study	-
6	1.2 Problem statement and rationale: Mentioned	-
7	1.3 Study objectives: Clearly outlined	-
8	1.4 Research methods	-
9	Study design: Appropriate	-
10	i) How was the study sites chosen? …authors mention that "these hospitals service regions with the highest rates of ARF in their respective countries" …are they representative of rest study population? …Any sampling procedure done? Q. Is the study cohort representative of all people presenting with ARF to hospital in AUS/NZ?	We have added: 'These hospitals service regions with the highest rates of ARF in their respective countries and were chosen to provide diversity in ethnicity and ARF severity.'<br>We note that Royal Darwin Hospital is a tertiary hospital in the Australia's Northern Territory; Kidz First Children's Hospital is a secondary hospital in Auckland, New Zealand; and Starship Children's Hospital is a quaternary hospital with specialist cardiothoracic services, also in Auckland, New Zealand.<br>Therefore, a mix of cases were anticipated at Royal Darwin, more severe cases requiring cardiac monitoring or intervention were anticipated at Starship Children's Hospital, and cases more representative of the wider population were anticipated at Kidz First Children's Hospital. Overall, this provided a range of ARF presentation types. In the absence of national registers at either site, we cannot comment on how representative the cohort is.
11	ii) The description of intervention is explicitly mentioned	-
12	iii) Inclusion criteria: Why differing age group of inclusion per study sites? i.e Royal Darwin Hospital (aged 5 to 30 years) vs Starship Hospital (aged 2 to 20 years). For Kidz First the included participants by age was not mentioned.	We have reworded to make it clearer that the age criteria were the same at the two New Zealand sites (added' Kidz First Hospital age criteria added (2 to 20 years)'). The different age profiles were agreed upon by local clinicals to reflect local epidemiology.
13	iv) Diagnostic process: The study intended to recruit participants up to age of 30yrs (eventually the oldest individual was 23 years) but there was no adult cardiologist	All adult echos were reported by an adult cardiologist. The treating clinician teams, and the research diagnostic panel, then used all available evidence including

	involved in the panel to review case record forms and primary data.	echo reports (+/- directly reviewing echo images where required) to assign diagnostic categories.<br>The clinical panels at the sites comprised clinicians routinely involved in the care of people with ARF at their respective sites, as well as research staff. At Royal Darwin Hospital for instance, adults with ARF are admitted under the General Medicine (adult Internal Medicine) unit, unless specific acute cardiological intervention is needed. In this study, of the ARF episodes, only 3 were in adults (aged 22, 22 and 23) and 140 in children. Therefore we are confident that the diagnostic review panel composition was appropriate.
14	v) Ethical clearance: Mentioned	-
15	1.5 Results: Nicely presented	-
16	1.6 Discussion: Authors report that “Useful information for people diagnosed with ARF and their families include details …”. Although it sound to be important information but it is not supported by the findings from the current study i.e was not researched on!	The rest of this sentence reads: ‘Useful information for people diagnosed with ARF and their families include details on anticipated hospital length of stay, how long NSAIDs and corticosteroids may need to be continued, and concomitant comorbidities such as nutritional issues (both over and undernutrition) that may need to be addressed among children presenting with ARF.’<br><br>We present detailed data on each of these elements:<br>-anticipated hospital length of stay: **Table 1, Table S9 and detailed in last paragraph of the results**<br>-how long NSAIDs and corticosteroids may need to be continued: **Data under subheading ‘Treatments received’ and Table S8**<br>- over and undernutrition: **Table 1, figure S3**<br><br>The Australian Indigenous Governance Group advised that the paper should include a focus on how the data

		collected from Aboriginal children can be of value for Aboriginal families. We are keen to retain this in the paper.
17	1.7 Conclusion: Authors argue that the study provided new information. As I previously commented, most of the information are not new. May be the author could say that their findings are proof of concept of what has been known from the past.	We respectfully differ on this point for the reasons provided in our response to comment #4.
18	1.8 Study limitations: Were clearly put forward	-
19	1.9 Dissemination of findings: Well planned	-
20	1.10 Conflict of interest: Authors declare no conflict of interest	-
21	1.11 Originality of Contribution: Relatively similar work has been done in the past.	-
22	1.12 Literature Citation: Good	-
23	1.13 Final Evaluation/Recommendation for Publication: Is worthy publication after responding to above comments	-
24	1.14 References: These are up-to-date citations	-
	Reviewer: 3	
25	Abstract: key words are missing	Added the following key words to the end of the abstract, as per the manuscript submission process: Cardiovascular, paediatrics, epidemiology, public health
26	Page 11, line 254: '118/143' should be episodes not participants. Participants were 141	Participants changed to ‘episodes’
27	Page 11, line 258: Clarify whether those are participants or episodes. The entire table 1 presents episodes and not total number of participants.	Participants changed to ‘episodes’
28	Page 11, line 261-266: Clarify whether 'cases' refers to number of patients or episodes.	‘Cases’ changed to ‘episodes’
29	Page 15, line 338-350: Clarify the presentation of RHD whether it is on number of patients or per ARF episodes. Table 1 presented only the number of episodes. Also, the total of 44 differs from 41 in table 1 and description at page 11.	‘Cases’ changed to ‘episodes’ throughout the manuscript.<br><br>Numbers in Table 1 and the text reconciled.
30	Page 16, line 349: Clarify whether the three participants are not included in the 41 as presented in table 1. This could be the difference between text on page 15 and page 11.	Numbers in Table 1 and the text reconciled.

1. Heart Foundation of New Zealand. New Zealand guidelines for rheumatic fever: diagnosis, management and secondary prevention of acute rheumatic fever and rheumatic heart disease: 2014 update. 2014.

2. Jack S, Moreland NJ, Meagher J, Fittock M, Galloway Y, Ralph AP. Streptococcal Serology in Acute Rheumatic Fever Patients: Findings From 2 High-income, High-burden Settings. *Pediatr Infect Dis J* 2019; **38**(1): e1-e6.

3. RHDAustralia (ARF/RHD writing group). The 2020 Australian guideline for prevention, diagnosis and management of acute rheumatic fever and rheumatic heart disease (3rd edition) https://www.rhdaustralia.org.au/arf-rhd-guideline. Menzies School of Health Research, 2020.
